# Ruthenium-Catalyzed *meta*-Selective C—H Bromination

**DOI:** 10.1002/anie.201504390

**Published:** 2015-08-18

**Authors:** Christopher J Teskey, Andrew Y W Lui, Michael F Greaney

**Affiliations:** School of Chemistry, The University of Manchester Oxford Road, Manchester, M13 9PL (UK)

**Keywords:** bromine, C–H activation, cross-coupling, regioselectivity, ruthenium

## Abstract

The first example of a transition-metal-catalyzed, *meta*-selective C–H bromination procedure is reported. In the presence of catalytic [{Ru(p-cymene)Cl_2_}_2_], tetrabutylammonium tribromide can be used to functionalize the *meta* C–H bond of 2-phenylpyridine derivatives, thus affording difficult to access products which are highly predisposed to further derivatization. We demonstrate this utility with one-pot bromination/arylation and bromination/alkenylation procedures to deliver *meta*-arylated and *meta*-alkenylated products, respectively, in a single step.

The field of catalytic C–H bond functionalization has grown significantly in recent years, thus offering new disconnections which can streamline synthetic routes and produce less waste.^1^ Several molecular architectures are now established for reliable C–H transformation, with arene C–H functionalization *ortho* to a directing group, by cyclometalation, being a prominent example.^2^ By contrast, *meta* functionalization is a more difficult reaction as the analogous cyclometalation processes are not at the chemists’ disposal. Given that stepwise *meta* functionalization is often challenging using classical arene chemistry, the development of new catalytic methods that address *meta* C–H functionality is of pressing importance.^3^ Several ground-breaking reaction systems have been developed to tackle this problem, principally in the areas of palladium and copper-catalyzed C–C bond formation,^4^–^8^ and iridium-catalyzed borylation.^9^ A third way of achieving *meta* functionalization has recently been described by the groups of Frost and Ackermann, where ruthenium catalysis is used for *meta* sulfonylation and alkylation, respectively.^10^ These reactions are thought to proceed by *ortho* ruthenation, thus affording an arylruthenium intermediate which exhibits a strong directing effect for functionalization at the C–H position *para* to the C–Ru bond.^11^ Addition of a suitable electrophile will thus result in overall *meta* substitution upon protonolysis of the C–Ru bond and completion of the catalytic cycle.

We were interested in exploring this concept in the context of *meta* bromination (Scheme 1). Aryl bromides are supremely versatile functional groups, with methods for C–H *ortho* bromination, and halogenation in general, undergoing extensive development in the C–H activation literature.^12^–^17^ However, *meta* bromination has yet to be described using transition-metal catalysis, and is restricted to very forcing reaction conditions in Friedel–Crafts bromination of electron-poor arenes (e.g., N–Br reagent in neat H_2_SO_4_ for bromination of nitrobenzene).^18^ A one-step *meta*-selective bromination, under mild reaction conditions, would open up a new pathway to valuable 1,3-bromo-functionalized arenes, which are currently prepared by tedious multistep routes. More generally, it would create a catalyst-controlled bromination system, where bromination of the same arene substrate could be directed to either the *ortho*- or *meta*-position depending upon the choice in catalyst.

**scheme 1:**
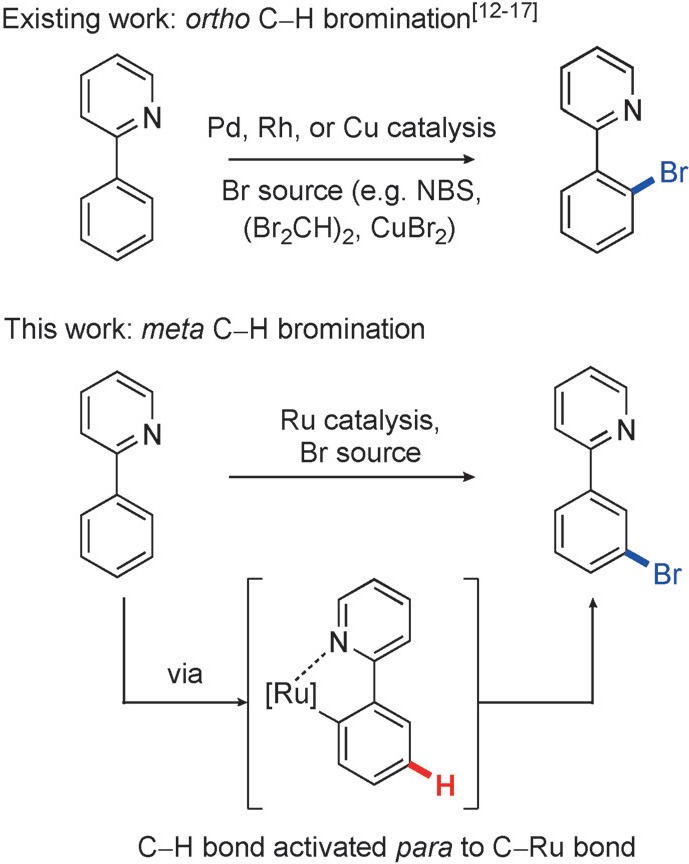
Transition-metal-catalyzed C–H bromination.

We began by screening electrophilic bromine sources in the presence of a base, catalytic [{Ru(*p*-cymene)Cl_2_}_2_], and 2-phenylpyridine (**1 a**), as the substrate. Initial results showed that NBS, bromine, and pyridinium tribromide gave minimal conversion to the desired *meta*-brominated product **2 a** (Table 1, entries 1–5). The failure of pyridinium tribromide is notable (entry 5) as this reagent has been successfully used to stoichiometrically brominate organo-ruthenium complexes.^11^ Gratifyingly, we observed successful *meta* bromination on switching to tetrabutylammonium tribromide (TBATB) in 1,4-dioxane, with **2 a** being formed with excellent conversion (entry 10). Use of a carboxylate additive in ruthenium catalyzed C–H activation chemistry has extensive precedent in work from the group of Ackermann,^19^ and acted in the current case to increase yields of the isolated products by 5–10 %. The reaction did not occur in the absence of ruthenium catalyst (entry 9) and in solvents other than 1,4-dioxane, no product was observed. Finally, the reaction was observed to be air-sensitive. In cases where the reaction was set up without rigorous removal of air, conversions were inconsistent but generally much lower.

**Table 1:**
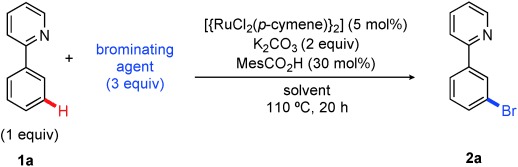
Reaction development.

Entry	Brominating agent	Solvent	1 a/2 a^[a]^
1	NBS	acetonitrile	>99:1
2	NBS	1,4-dioxane	>95:5
3	Br_2_	acetonitrile	>99:1
4	Br_2_	1,4-dioxane	>99:1
5	pyridinium tribromide	1,4-dioxane	>99:1
6	TBATB	acetonitrile	>99:1
7	TBATB	water	>99:1
8^[b]^	TBATB	1,4-dioxane	10:90
9^[c]^	TBATB	1,4-dioxane	>99:1
10	TBATB	1,4-dioxane	5:95

[a] Ratio of **1 a**/**2 a** is based on ^1^H NMR analysis of crude reaction mixtures after work-up.

[b] Reaction carried out without MesCO_2_H additive.

[c] Experiment carried out without ruthenium catalyst.

With the optimized reaction conditions in hand, we sought to explore the substrate scope (Scheme 2). We were pleased to find that both electron-donating (**2 b**–**d**) and electron-withdrawing groups (**2 e**–**g**) in the *para*-position of the aromatic ring were well tolerated, producing good to excellent yields of the bromide. In cases where the *para*-substituent possesses significant steric bulk the reaction still proceeds, but at a slower rate, thus resulting in a low yield after 20 hours (**2 d**). It should be noted that in low-yielding cases, the majority of the remaining material can be accounted for as starting the 2-phenylpyridine substrate. The reaction is remarkably selective for the monobrominated, rather than the dibrominated, product, despite using an excess of brominating agent. Over-bromination has been problematic in some previous examples of metal-catalyzed *ortho* bromination.13b, 15a The selectivity obtained in the *meta* bromination relative to other transition-metal-catalyzed bromination methods is exemplified by the reaction of benzo[h]quinoline to give the 7-brominated compound **2 j**. This product could not be obtained selectively by using existing bromination methods,^20^ and it contains a new C–Br bond at a useful site for further modification. Functionalized benzoquinolines are used extensively as ligands in areas such as photoredox catalysis, metallo-supramolecular chemistry, and organic electronics, where methods for modifying the ligand structure are essential to fine-tune electronic properties.

**scheme 2:**
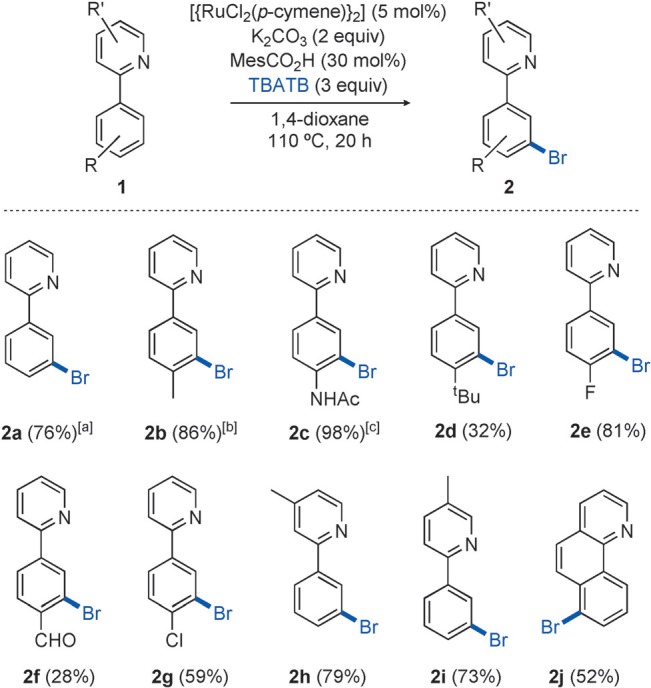
Substrate scope for *meta* bromination. Yields are those of isolated products. [a] Average of three runs. [b] Average of two runs. [c] Yield without ruthenium catalyst is 10 %. NBS=*N*-bromosuccinimide.

As with the sulfonylation system reported by Frost and co-workers,10a *meta* substitution on the phenyl ring is not tolerated. Ruthenation is presumably directed to the most sterically accessible *ortho* position, meaning that the pre-existing *meta* substituent is now blocking the site of bromination. Likewise, *ortho* substitution was not tolerated in phenylpyridine substrates. It is likely that this additional steric encumbrance prevents co-planarity of the phenyl–pyridine biaryl, thus disrupting the directed metalation and ensuing bromination. However, with substitution at other positions on the pyridine directing group, the reaction proceeds in excellent yield (**2 h**,**i**). Pleasingly, we were able to scale-up the reaction to a 5 mmol scale. By running the reaction for 65 hours, but with half the catalyst loading (2.5 mol %), the yield of isolated **2 a** remained at 76 %, with 4 % of the *ortho*-brominated product also isolated.

To demonstrate the versatility of this methodology, we developed simple one-pot processes to further manipulate the newly installed bromide group in C–C bond-forming reactions (Scheme 3).^21^ We could *meta*-arylate by a one-pot bromination/Suzuki–Miyaura coupling: additional base was used in the first step, and after running the bromination for 20 hours, water, Pd(OAc)_2_ (3 mol %), PPh_3_ (6 mol %), and either a boronic acid or ester (3 equiv) were added and the reaction run for a further 15 hours. This one-pot *meta*-arylation procedure worked well for *ortho*-, *meta*-, and *para*- substituted boronic acids, and both electron-withdrawing and electron-donating substituents were tolerated (**3 a**–**e**). The reaction was extended to heteroaromatic boronic esters, with the use of *N*-Boc-pyrrole-2-boronic acid MIDA ester proving effective for the synthesis of **3 f** in 64 % yield. A *meta*-alkenylation process was also possible: by simply adding Pd(OAc)_2_ (3 mol %) and three equivalents of a suitable alkene, post-bromination, and heating the reaction to 110 °C a one-pot bromination/Heck reaction proceeded. Yields of the alkenylated product over the two steps were good (**4 a** and **4 c**), and the use of but-3-en-2-ol gave the alkylated ketone product **4 b** in 55 % yield.

**scheme 3:**
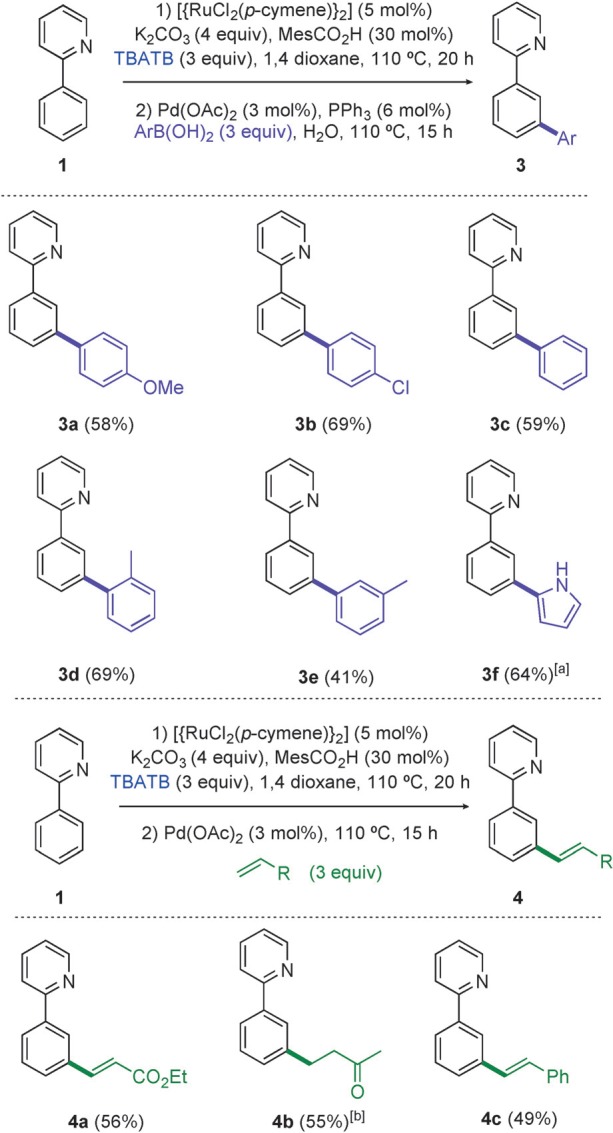
Substrate scope for one-pot transformations. Yields are those of isolated products. [a] *N*-Boc-pyrrole-2-boronic acid MIDA ester used as boronic acid starting material. [b] But-3-en-2-ol used as olefin starting material. MIDA=*N*-methyliminodiacetic acid.

Finally, we could successfully convert the pyridine directing group into the saturated heterocycle **5** (Scheme 4). Pyridine reduction is a versatile entry point into functionalized piperidines, which are heavily exploited scaffolds in medicinal chemistry. Here, treatment of **2 a** with SmI_2_/H_2_O rapidly reduced the heteroarene,^22^ thus leaving the aryl bromide group intact for further manipulation.

**scheme 4:**
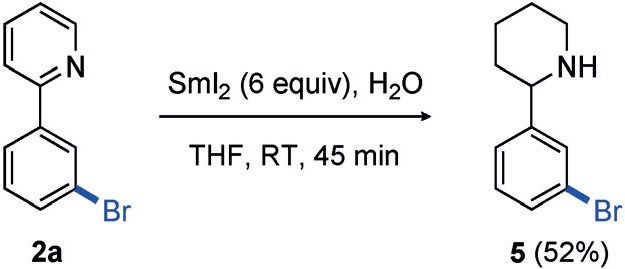
Reduction of pyridine directing group using SmI_2_. THF= tetrahydrofuran.

To conclude, we report the first example of transition-metal-catalyzed *meta*-selective bromination. The orthogonal selectivity exhibited by ruthenium relative to copper, palladium, and rhodium catalysis offers a catalyst-controlled route to compounds which may have been previously difficult to synthesize. Further, the reaction system is amenable to one-pot, telescoped processes which enable *meta* arylation and *meta* alkenylation. Further investigations into the scope of this chemistry are currently underway in our laboratory.

## Supporting Information

As a service to our authors and readers, this journal provides supporting information supplied by the authors. Such materials are peer reviewed and may be re-organized for online delivery, but are not copy-edited or typeset. Technical support issues arising from supporting information (other than missing files) should be addressed to the authors.

miscellaneous_information
