# Supplementary material for: Ruthenium-Catalyzed meta-Selective C—H Bromination
Source: Angew Chem Int Ed Engl. 2015 Aug 18;54(40):11677–80. doi: 10.1002/anie.201504390 (PMC4678425; doi:10.1002/anie.201504390)

Angewandte Chemie
Eine Zeitschrift der Gesellschaft Deutscher Chemiker

Supporting Information

Ruthenium-Catalyzed *meta*-Selective C-H Bromination

*Christopher J. Teskey, Andrew Y. W. Lui, and Michael F. Greaney**

anie_201504390_sm_miscellaneous_information.pdf

Supporting Information

General remarks

Nuclear Magnetic Resonance (NMR) spectra were recorded on 500 or 400 MHz Bruker NMR spectrometers in $CDCl_3$ at 298 K (unless stated otherwise). All chemical shift values are reported in parts per million (ppm) relative to the solvent signal (^{1}H NMR: δ = 7.26 ppm, ^{13}C NMR: δ = 77.16 ppm) with coupling constant (J) values reported to the nearest 0.1 Hz. Splitting patterns are abbreviated as follows: singlet (s), doublet (d), triplet (t), quartet (q), quintet (quin.), multiplet (m), broad singlet (br s) and combinations thereof.

Reactions were carried out under N_2 using pre-dried glassware which was cooled under vacuum. All reactions were carried out in schlenk tubes purchased from Sigma Aldrich and heated in oil baths with a thermocouple temperature control. 1,4-dioxane was purchased in anhydrous quality and used as received. Reagents were either purchased directly from commercial suppliers or prepared according to literature procedures. Potassium carbonate was dried under vacuum before being weighed. Yields of all the compounds refer to isolated compounds. TLC: Macherey-Nagel, TLC plates Alugram® Sil G/UV254. Detection under UV light at 254 nm. Chromatography: Separations were carried out on Silica gel (Sigma Aldrich, 40-63 µm, 60 Å). High resolution mass spectrometry was performed on a Waters QTOF with ESI/APCI ionisation and a Thermo Finnigan MAT95XP (EI). Melting points were determined using a Buchi M565 melting point apparatus. SmI_2 was prepared according to literature procedure.[1]

Substrates for the *meta*-bromination were typically synthesised using the following procedure: To the boronic acid (3.0 mmol, 1.5 eq), palladium acetate (6.7 mg, 0.030 mmol, 0.015 eq) and potassium carbonate (553 mg, 4.0 mmol, 2.0 eq) was added ethanol (9 ml), water (3 ml) and 2-bromopyridine (191.5 µl, 2.0 mmol, 1.0 eq). After heating at 80 ºC for 20 hours, water was added and the mixture extracted three times with ethyl acetate. The combined organic layers were washed with brine, concentrated and purified by flash column chromatography (ethyl acetate, hexane mixtures). All data for the synthesised compounds was in accordance with known literature values.

Reaction discovery and optimisation

Ratios of starting material to product were taken from the ^{1}H NMR spectrum of the crude reaction mixture after standard work up procedure.

Brominating agent

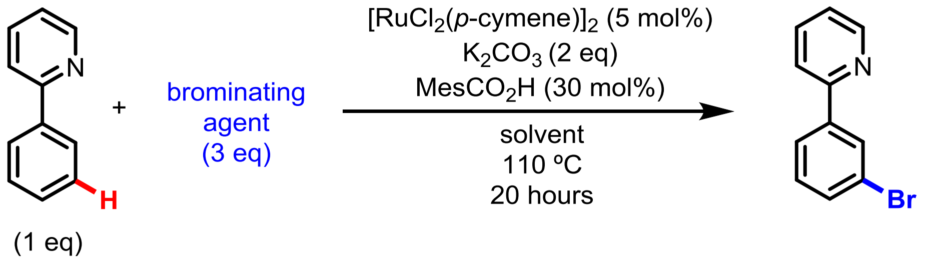

1a ***2a***

Entry	Brominating agent	Solvent	*1a:2a*
**1**	NBS	MeCN	>99:1
**2**	NBS	1,4-dioxane	>95:5
**3**	Br_2	MeCN	>99:1
**4**	Br_2	1,4-dioxane	>99:1
**5**	TBATB	MeCN	>99:1
**6**	TBATB	1,4-dioxane	5:95

Ruthenium loading

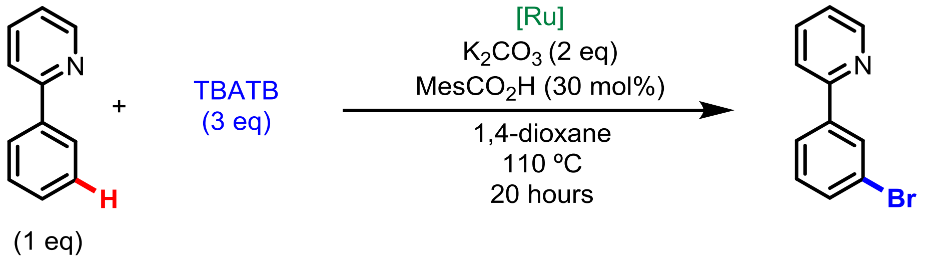

1a ***2a***

Entry	[Ru]	*1a:2a*
**1**	$[RuCl_2(p\text{-cymene})]_2$ (5 mol%)	5:95
**2**	$[RuCl_2(p\text{-cymene})]_2$ (2.5 mol%)	40:60
**3**	$[RuCl_2(p\text{-cymene})]_2$ (1 mol%)	70:30
**4**	-	100:0
**5**	$Ru_3(CO)_{12}$ (3 mol%)	90:10

Solvent screen

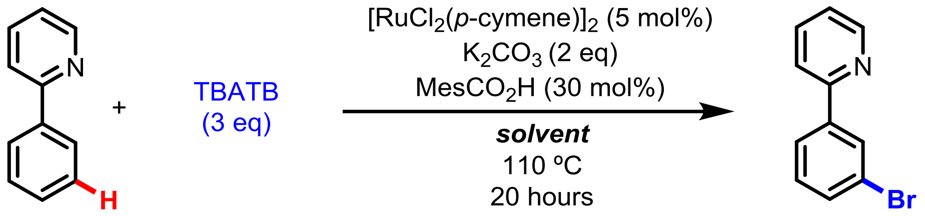

(1 eq)

1a *2a*

Entry	Solvent	*1a:2a*
**1**	MeCN	>99:1
**2**	DMF	>99:1
**3**	Water	>99:1
**4**	1,4-dioxane/water (1:1)	>99:1
**5**	1,4-dioxane	5:95

Temperature and TBATB loading

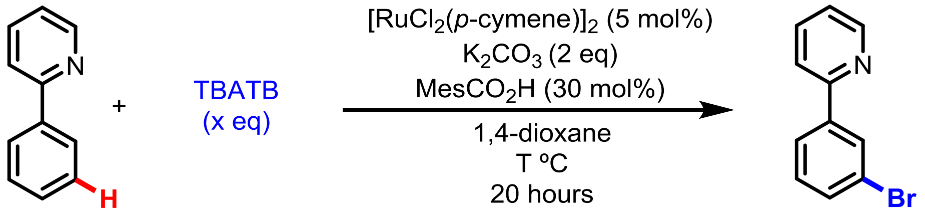

(1 eq)

1a *2a*

Entry	(x eq)	T °C	*1a:2a*
**1**	2	rt	100:0
**2**	2	90	60:40
**3**	2	110	30:70
**4**	3	110	5:95

Control experiments

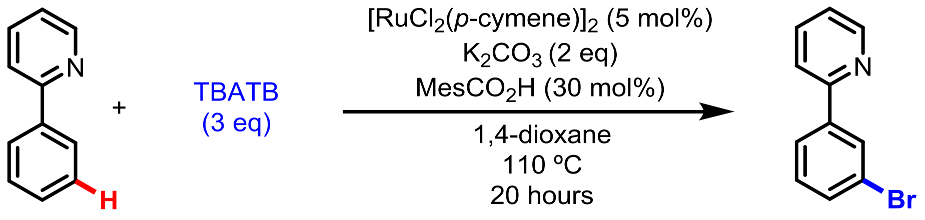

(1 eq)

1a *2a*

Entry	$[RuCl_2(p\text{-cymene})]_2$	K_2CO_3	$MesCO_2H$	*1a:2a*
**1**	N	Y	Y	100:0
**2**	Y	N	Y	50:50
**3**	Y	Y	N	10:90
**4**	Y	Y	Y	5:95

Experimental procedures and data

Bromination procedure

A schlenk tube was charged with $[RuCl_2(p\text{-cymene})]_2$ (15.3 mg, 0.025 mmol, 5 mol%), potassium carbonate (138 mg, 1.0 mmol, 2.0 eq), $MesCO_2H$ (24.6 mg, 0.15 mmol, 0.3 eq) and TBATB (723 mg, 1.5 mmol, 3.0 eq) before being evacuated and then back-filled with nitrogen three times. Dry 1,4-dioxane (2 ml) was added and then 2-phenylpyridine (71.5 µl, 0.5 mmol, 1.0 eq) in dioxane (1 ml) before the flask was sealed and the reaction mixture heated to 110 ºC for 20 hours. After cooling, sodium thiosulphate (~20 ml, 10 wt%) was added before extracting the aqueous layer with EtOAc (× 3). The combined organic layers were washed with brine and then dried over $MgSO_4$. After concentration *in vacuo*, the crude reaction mixture was purified by flash column chromatography (typically ethyl acetate:hexane (2:98 to 10:90) although dichloromethane:hexane mixtures were used in some cases where separation from starting material was much clearer).

2-(3-bromophenyl)pyridine[2], *2a*

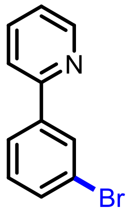

Yellow oil, average of three yields: 76%; ^1H NMR (400 MHz, $CDCl_3$): δ 8.72 (d, *J* = 4.8 Hz, 1H), 8.20 (*app* t, *J* = 1.5 Hz, 1H), 7.93 (d, *J* = 7.9 Hz, 1H), 7.79 (*app* td, *J* = 7.5, 1.5 Hz, 1H), 7.72 (d, *J* = 8.0 Hz, 1H), 7.56 (d, *J* = 7.9 Hz, 1H), 7.36 (t, *J* = 8.0 Hz, 1H), 7.30 – 7.26 (m, 1H); ^{13}C NMR (100 MHz, $CDCl_3$): δ 156.0, 149.9, 141.5, 137.0, 132.0, 130.4, 130.2, 125.5, 123.2, 122.8, 120.7; HRMS (EI): calculated for $C_{11}H_8NBr$, theoretical 232.9835, measured 232.9831.

2-(3-bromo-4-methylphenyl)pyridine, *2b*

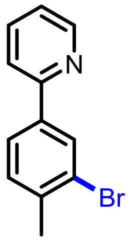

Yellow oil, average of two yields: 86%; ^1H NMR (400 MHz, $CDCl_3$): δ 8.68 (d, *J* = 4.7 Hz, 1H), 8.20 (d, *J* = 1.4 Hz, 1H), 7.83 (dd, *J* = 7.9, 1.4 Hz, 1H), 7.76 – 7.72 (m, 1H), 7.68 (d, *J* = 7.9 Hz, 1H), 7.32 (d, *J* = 8.0 Hz, 1H), 7.25 – 7.22 (m, 1H), 2.45 (s, 3H); ^{13}C NMR (100 MHz, $CDCl_3$): δ 156.0, 149.8, 138.9, 138.8, 137.0, 131.2, 130.8, 125.7, 125.6, 122.5, 120.4, 22.9; HRMS (EI): calculated for $C_{12}H_{10}NBr$, theoretical 246.9991, measured 246.9993.

N-(2-bromo-4-(pyridin-2-yl)phenyl)acetamide, *2c*

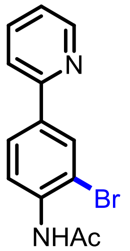

Off- white solid, 143.1 mg, 98%. %; ^1H NMR (400 MHz, $CDCl_3$): δ 8.67 (m, 1H), 8.48 (d, *J* = 8.6 Hz, 1H), 8.30 (d, *J* = 2.1 Hz, 1H), 7.90 (dd, *J* = 8.6, 2.1 Hz, 1H), 7.74 (m, 1H), 7.72 (br s, 1H), 7.69 (m, 1H), 7.74 (m, 1H), 2.27 (s, 3H); ^{13}C NMR (100 MHz, $CDCl_3$): 168.4, 155.3, 149.8, 137.0, 136.3, 136.2, 130.7, 126.7, 122.5, 121.6, 120.3, 113.7, 25.1. HRMS (EI): calculated for $C_{13}H_{11}BrN_2O$, theoretical 290.0049, measured 290.0047; m.p. 132 – 135 ºC.

2-(3-bromo-4-(*tert*-butyl)phenyl)pyridine, *2d*

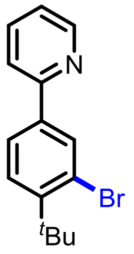

Yellow oil, 47.0 mg, 32%; ^{1}H NMR (400 MHz, $CDCl_3$): δ 8.71 – 8.69 (m, 1H), 7.75 (*app* td, J = 7.7, 1.8 Hz, 1H), 7.67 (d, J = 1.8 Hz, 1H), 7.61 (*app* dt, J = 7.9, 1.0 Hz, 1H), 7.48 (d, J = 8.1 Hz, 1H), 7.42 (dd, J = 8.1, 1.8 Hz, 1H), 7.29 – 7.26 (m, 1H), 1.34 (s, 9H); ^{13}C NMR (100 MHz, $CDCl_3$): δ 158.4, 153.5, 149.4, 138.3, 135.9, 131.2, 130.4, 124.9, 124.9, 122.4, 121.7, 34.9, 31.3; HRMS (EI): calculated for $C_{15}H_{16}NBr$, theoretical 289.0461, measured 289.0451.

2-(3-bromo-4-fluorophenyl)pyridine, *2e*

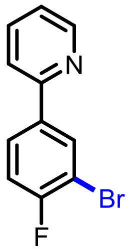

White solid, 102.0 mg, 81%; ^{1}H NMR (400 MHz, $CDCl_3$): δ 8.68 (d, J = 4.4 Hz, 1H), 8.24 (dd, J = 6.7, 2.1 Hz, 1H), 7.92 – 7.88 (m, 1H), 7.78 – 7.74 (m, 1H), 7.67 (d, J = 8.0 Hz, 1H), 7.27 – 7.24 (m, 1H), 7.21 (*app* t, J = 8.4 Hz, 1H); ^{13}C NMR (100 MHz, $CDCl_3$): δ 159.8 (d, $J_{C\text{-}F}$ = 249.6 Hz), 155.1, 149.9, 137.1, 137.0 (d, $J_{C\text{-}F}$ = 3.7 Hz), 132.3, 127.5 (d, $J_{C\text{-}F}$ = 7.6 Hz), 122.7, 120.4, 116.8 (d, J = 22.5 Hz), 109.7 (d, J = 21.3 Hz); ^{19}F NMR (376 MHz, $CDCl_3$): δ -104.22 – -110.57 (m); HRMS (APCI+): calculated for $C_{11}H_8NBrF$ (M + H^+), theoretical 251.9824, measured 251.9835; m.p. 47 – 49 ºC.

2-bromo-4-(pyridin-2-yl)benzaldehyde, *2f*

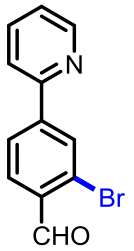

Off-white solid, 36.7 mg, 28%; ^{1}H NMR (400 MHz, $CDCl_3$): δ 10.41 (s, 1H), 8.76 – 8.70 (m, 1H), 8.37 (d, J = 1.4 Hz, 1H), 8.07 – 7.97 (m, 2H), 7.86 – 7.73 (m, 2H), 7.34 (ddd, J = 6.7, 4.8, 1.7 Hz, 1H); ^{13}C NMR (100 MHz, $CDCl_3$): δ 191.8, 154.5, 150.3, 146.0, 137.3, 133.4, 132.4, 130.3, 127.77, 126.2, 123.8, 121.4; HRMS (APCI+), calculated for $C_{12}H_9NOBr$ (M + H^+), theoretical 261.9868, measured 261.9863; m.p. 95 – 97 ºC.

2-(3-bromo-4-chlorophenyl)pyridine, *2g*

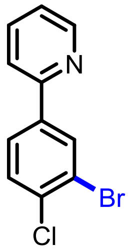

Off-white solid, 79.0 mg, 59%; ^{1}H NMR (400 MHz, $CDCl_3$): δ 8.69 (d, J = 4.3 Hz, 1H), 8.29 (d, J = 2.1 Hz, 1H), 7.87 (dd, J = 8.3, 2.1 Hz, 1H), 7.79 – 7.74 (m, 1H), 7.69 – 7.67 (m, 1H), 7.53 (d, J = 8.3 Hz, 1H), 7.28 – 7.25 (m, 1H); ^{13}C NMR (100 MHz, $CDCl_3$): δ 154.9, 150.0, 139.4, 137.2, 135.2, 132.2, 130.6, 126.8, 123.1, 123.0, 120.5; HRMS (EI): calculated for $C_{11}H_7ClBrN$, theoretical 266.9445, measured 266.9455; m.p. 60 – 62 ºC.

2-(3-bromophenyl)-4-methylpyridine[3], *2h*

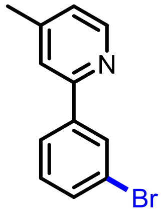

Yellow oil, 98.0 mg, 79%; ^{1}H NMR (400 MHz, $CDCl_3$): δ 8.54 (dd, J = 5.0, 0.8 Hz, 1H), 8.15 (*app* t, J = 1.9 Hz, 1H), 7.88 (*app* dt, J = 7.9, 1.4 Hz, 1H), 7.55 – 7.49 (m, 2H), 7.33 (*app* t, J = 7.9 Hz, 1H), 7.12 – 7.06 (m, 1H), 2.42 (s, 3H); ^{13}C NMR (100 MHz, $CDCl_3$): δ 155.8, 149.6, 148.1, 141.6, 131.8, 130.3, 130.1, 125.5, 123.8, 123.1, 121.7, 21.4; HRMS (APCI+), calculated for $C_{12}H_{11}NBr$ (M + H^+), theoretical 248.0075, measured 248.0083.

2-(3-bromophenyl)-5-methylpyridine[4], *2i*

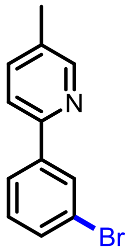

Yellow oil, 90.6 mg, 73%; ^{1}H NMR (400 MHz, $CDCl_3$): δ 8.52 (m, 1H), 8.14 (*app* t, J = 1.8 Hz, 1H), 7.88 (m, 1H), 7.60 (dd, J = 8.1, 1.0 Hz, 1H), 7.56 (m, 1H), 7.51 (m, 1H), 7.32 (*app* t, J = 8.0 Hz, 1H), 2.38 (s, 3H); ^{13}C NMR (100 MHz, $CDCl_3$): δ 153.3, 150.4, 141.6, 137.6, 132.5, 131.6, 130.3, 129.9, 125.3, 123.2, 120.2, 18.4.; HRMS (EI): calculated for $C_{12}H_{10}NBr$, theoretical 246.9991, measured 246.9986.

7-bromobenzo[h]quinolone, *2j*

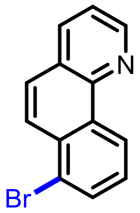

Off-white solid, 67.4 mg, 52%; ^{1}H NMR (400 MHz, $CDCl_3$): δ 9.34 – 9.30 (m, 1H), 9.02 (dd, J = 4.3, 1.8 Hz, 1H), 8.24 (d, J = 9.1 Hz, 1H), 8.18 (dd, J = 8.0, 1.8 Hz, 1H), 7.97 (dd, J = 7.5, 1.2 Hz, 1H), 7.76 (d, J = 9.1 Hz, 1H), 7.60 – 7.52 (m, 2H); ^{13}C NMR (100 MHz, $CDCl_3$): δ 149.5, 146.1, 136.1, 133.3, 132.4, 132.2, 127.6, 126.9, 126.3, 126.2, 124.3, 122.9, 122.5; HRMS (EI): calculated for $C_{13}H_8NBr$, theoretical 256.9835, measured 256.9830; m.p. 84 – 86 ºC.

One-pot bromination-Suzuki-Miyaura reactions

A schlenk tube was charged with $[RuCl_2(p\text{-cymene})]_2$ (15.3 mg, 0.025 mmol, 5 mol%), potassium carbonate (276 mg, 2.0 mmol, 4.0 eq), $MesCO_2H$ (24.6 mg, 0.15 mmol, 0.3 eq) and TBATB (723 mg, 1.5 mmol, 3.0 eq) before being evacuated and then back-filled with nitrogen three times. Dry 1,4-dioxane (2 ml) was added and then 2-phenylpyridine (71.5 µl, 0.5 mmol, 1.0 eq) in dioxane (1 ml) before the flask was sealed and the reaction mixture heated to 110 ºC for 20 hours. After cooling, water (1 ml), $Pd(OAc)_2$ (3.4 mg, 0.015 mmol, 3 mol%), PPh_3 (7.9 mg, 0.03 mmol, 6 mol%) and the boronic acid (1.5 mmol, 3.0 eq) were added under a stream of nitrogen. After resealing the vial and heating at 110 ºC for 15 hours, the reaction mixture was allowed to cool and sodium thiosulphate (~20 ml, 10 wt%) was added. The reaction mixture was then extracted with EtOAc (× 3). The combined organic layers were washed with brine and then dried over $MgSO_4$. After concentration *in vacuo*, the crude reaction mixture was purified by flash column chromatography (typically ethyl acetate:hexane (4:96 to 20:80)).

2-(4'-methoxy-[1,1'-biphenyl]-3-yl)pyridine, *3a*

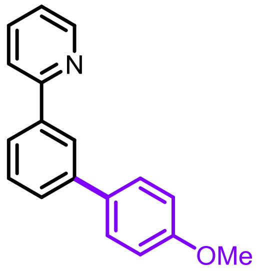

Yellow solid, 75.5 mg, 58%; ^{1}H NMR (400 MHz, $CDCl_3$): 8.75 – 8.70 (m, 1H), 8.21 (*app* t, J = 1.9 Hz, 1H), 7.92 (ddd, J = 7.6, 1.8, 1.2 Hz, 1H), 7.81 – 7.73 (m, 2H), 7.65 – 7.59 (m, 3H), 7.53 (*app* t, J = 7.7 Hz, 1H), 7.27 – 7.21 (m, 1H), 7.03 – 6.96 (m, 2H), 3.86 (s, 3H); ^{13}C NMR (100 MHz, $CDCl_3$): δ 159.4, 157.7, 149.8, 141.5, 140.0, 136.9, 133.7, 129.3, 128.4, 127.5, 125.6, 125.4, 122.3, 120.9, 114.3, 55.5; HRMS (APCI+) calculated for $C_{18}H_{16}NO$ $(M + H^+)$, theoretical 262.1232, measured 262.1237; m.p. 65 – 67 ºC.

2-(4'-chloro-[1,1'-biphenyl]-3-yl)pyridine, *3b*

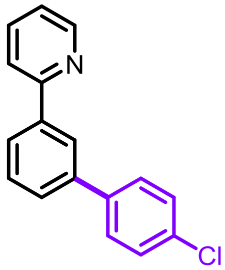

Yellow oil, 91.2 mg, 69%; ^{1}H NMR (400 MHz, $CDCl_3$): δ 8.76 – 8.69 (m, 1H), 8.21 (*app* t, *J* = 1.7 Hz, 1H), 7.96 (dd, *J* = 7.7, 1.5 Hz, 1H), 7.79 (d, *J* = 4.1 Hz, 2H), 7.63 – 7.58 (m, 3H), 7.55 (*app* t, *J* = 7.7 Hz, 1H), 7.43 (d, *J* = 8.3 Hz, 2H), 7.29 – 7.24 (m, 1H); ^{13}C NMR (100 MHz, $CDCl_3$): δ 157.2, 149.8, 140.6, 140.1, 139.5, 136.9, 133.6, 129.4, 129.0, 128.6, 127.6, 126.2, 125.7, 122.5, 120.8; HRMS (APCI+), calculated for $C_{17}H_{13}NCl$ (M + H^+), theoretical 266.0737, measured 266.0736.

2-([1,1'-biphenyl]-3-yl)pyridine[5], *3c*

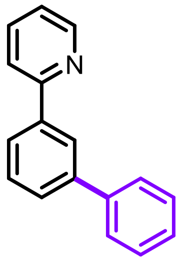

Yellow oil, 68.7 mg, 59%; ^{1}H NMR (500 MHz, $CDCl_3$): δ 8.75 – 8.70 (m, 1H), 8.25 (*app* t, *J* = 1.7 Hz, 1H), 7.99 – 7.95 (m, 1H), 7.81 – 7.75 (m, 2H), 7.71 – 7.68 (m, 2H), 7.67 – 7.64 (m, 1H), 7.56 (*app* t, *J* = 7.7 Hz, 1H), 7.47 (*app* t, *J* = 7.7 Hz, 2H), 7.38 (*app* tt, *J* = 7.2, 1.4 Hz, 1H), 7.27 – 7.23 (m, 1H); ^{13}C NMR (125 MHz, $CDCl_3$): δ 157.5, 149.8, 141.9, 141.2, 140.0, 136.9, 129.3, 128.9, 127.9, 127.5, 127.4, 126.0, 126.0, 122.4, 120.8.; HRMS (APCI+), calculated for $C_{17}H_{14}N$ (M + H^+), theoretical 232.1126, measured 232.1120.

2-(2'-methyl-[1,1'-biphenyl]-3-yl)pyridine, *3d*

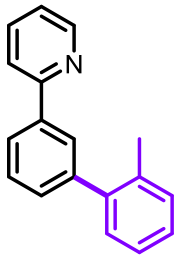

Yellow oil, 84.1 mg, 69%; ^{1}H NMR (400 MHz, $CDCl_3$): δ 8.73 – 8.68 (m, 1H), 7.99 (ddd, *J* = 7.8, 1.9, 1.2 Hz, 1H), 7.95 (*app* t, *J* = 1.7 Hz, 1H), 7.78 – 7.72 (m, 2H), 7.53 (*app* t, *J* = 7.7 Hz, 1H), 7.39 (*app* dt, *J* = 7.6, 1.4 Hz, 1H), 7.31 – 7.22 (m, 5H), 2.29 (s, 3H); ^{13}C NMR (100 MHz, $CDCl_3$): δ 157.5, 149.8, 142.5, 141.8, 139.4, 136.9, 135.5, 130.4, 129.9, 129.9, 128.6, 127.9, 127.5, 125.9, 125.5, 122.3, 120.8, 20.7; HRMS (APCI+), calculated for $C_{18}H_{16}N$ (M + H^+), theoretical 246.1283, measured 246.1278.

2-(3'-methyl-[1,1'-biphenyl]-3-yl)pyridine, *3e*

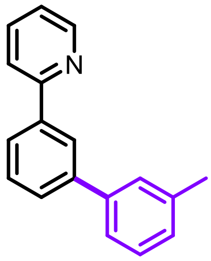

Yellow oil, 50.8 mg, 41%; ^{1}H NMR (500 MHz, $CDCl_3$): 8.77 – 8.69 (m, 1H), 8.22 (*app* t, *J* = 1.8 Hz, 1H), 8.02 – 7.88 (m, 1H), 7.82 – 7.75 (m, 2H), 7.68 – 7.62 (m, 1H), 7.55 (*app* t, *J* = 7.7 Hz, 1H), 7.52 – 7.46 (m, 2H), 7.36 (*app* t, *J* = 7.6 Hz, 1H), 7.28 – 7.24 (m, 1H), 7.21 – 7.17 (m, 1H), 2.44 (s, 3H); ^{13}C NMR (125 MHz, $CDCl_3$): δ 157.6, 149.8, 142.0, 141.2, 140.0, 138.5, 137.0, 129.3, 128.8, 128.3, 128.2, 127.9, 126.0, 125.9, 124.5, 122.4, 120.9, 21.7; HRMS (APCI+) calculated for $C_{18}H_{15}N$ (M + H^+), theoretical 246.1283, measured 246.1271.

2-(3-(1H-pyrrol-2-yl)phenyl)pyridine, *3f*

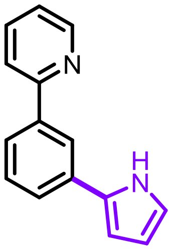

Brown oil, 70.6 mg, 64%; ^{1}H NMR (400 MHz, $CDCl_3$): δ 8.93 (br s, 1H), 8.76 – 8.64 (m, 1H), 8.14 (*app* t, *J* = 1.8 Hz, 1H), 7.79 – 7.71 (m, 3H), 7.55 (*app* dt, *J* = 8.0, 1.4 Hz, 1H), 7.44 (*app* t, *J* = 7.7 Hz, 1H), 7.29 – 7.23 (m, 1H), 6.88 – 6.84 (m, 1H), 6.63 – 6.58 (m, 1H), 6.34 – 6.30 (m, 1H); ^{13}C NMR (100 MHz, $CDCl_3$): δ 157.4, 149.7, 139.9, 137.1, 133.4, 132.0, 129.4, 124.8, 124.7, 122.5, 122.3, 120.9, 119.2, 110.1, 106.4; HRMS (APCI): calculated for $C_{15}H_{13}N_2$ (M + H^+), theoretical 221.1079, measured 221.1077.

One-pot bromination-Heck reactions

A schlenk tube was charged with $[RuCl_2(p\text{-cymene})]_2$ (15.3 mg, 0.025 mmol, 5 mol%), potassium carbonate (276 mg, 2.0 mmol, 4.0 eq), $MesCO_2H$ (24.6 mg, 0.15 mmol, 0.3 eq) and TBATB (723 mg, 1.5 mmol, 3.0 eq) before being evacuated and then back-filled with nitrogen three times. Dry 1,4-dioxane (2 ml) was added and then 2-phenylpyridine (71.5 µl, 0.5 mmol, 1.0 eq) in dioxane (1 ml) before the flask was sealed and the reaction mixture heated to 110 ºC for 20 hours. After cooling, $Pd(OAc)_2$ (3.4 mg, 0.015 mmol, 3 mol%) and the olefin (1.5 mmol, 3.0 eq) were added under a stream of nitrogen. After resealing the vial and heating at 110 ºC for 15 hours, the reaction mixture was allowed to cool and sodium thiosulphate (~20 ml, 10 wt%) was added. The reaction mixture was then extracted with EtOAc (× 3). The combined organic layers were washed with brine and then dried over $MgSO_4$. After concentration *in vacuo*, the crude reaction mixture was purified by flash column chromatography (typically ethyl acetate:hexane (4:96 to 20:80))

ethyl (E)-3-(3-(pyridin-2-yl)phenyl)acrylate, *4a*

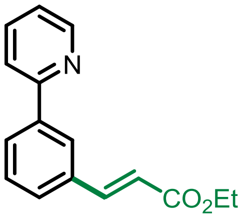

Yellow solid, 71.4 mg, 56%; ^{1}H NMR (400 MHz, $CDCl_3$): δ 8.74 – 8.67 (m, 1H), 8.18 (*app* t, *J* = 1.8 Hz, 1H), 8.00 – 7.97 (m, 1H), 7.80 – 7.71 (m, 3H), 7.57 (*app* dt, *J* = 7.8, 1.5 Hz, 1H), 7.49 (*app* t, *J* = 7.7 Hz, 1H), 7.28 – 7.24 (m, 1H), 6.55 (d, *J* = 16.0 Hz, 1H), 4.27 (q, *J* = 7.1 Hz, 2H), 1.34 (t, *J* = 7.1 Hz, 3H); ^{13}C NMR (100 MHz, $CDCl_3$): δ 167.0, 156.6, 149.8, 144.4, 140.1, 136.9, 135.0, 129.3, 128.6, 128.5, 126.7, 122.6, 120.6, 118.8, 60.6, 14.4; HRMS (EI): calculated for $C_{16}H_{15}NO_2$, theoretical 253.1097, measured 253.1101; m.p. 63 – 65 ºC.

4-(3-(pyridin-2-yl)phenyl)butan-2-one, *4b*

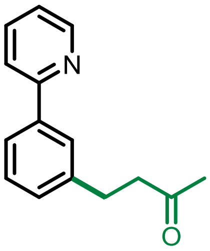

Yellow oil, 61.7 mg, 55%; ^{1}H NMR (400 MHz, $CDCl_3$): δ 8.70 (dd, *J* = 4.7, 1.5 Hz, 1H), 7.86 (*app* t, *J* = 1.8 Hz, 1H), 7.81 – 7.68 (m, 3H), 7.39 (*app* t, *J* = 7.7 Hz, 1H), 7.29 – 7.21 (m, 2H), 2.99 (t, *J* = 7.6 Hz, 2H), 2.83 (t, *J* = 7.6 Hz, 2H), 2.16 (s, 3H); ^{13}C NMR (100 MHz, $CDCl_3$): δ 208.1, 157.5, 149.7, 141.7, 139.7, 137.0, 129.2, 129.1, 127.0, 124.9, 122.3, 120.9, 45.4, 30.3, 29.9; HRMS (APCI+): calculated for $C_{15}H_{16}NO$ (M + H^+), theoretical 226.1232, measured 226.1225.

(E)-2-(3-styrylphenyl)pyridine, *4c*

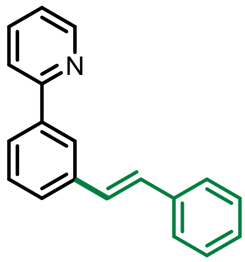

Yellow solid, 63.0 mg, 49%; ^{1}H NMR (400 MHz, $CDCl_3$): δ 8.75 – 8.70 (m, 1H), 8.18 (*app* t, *J* = 1.8 Hz, 1H), 7.89 – 7.82 (m, 1H), 7.81 – 7.76 (m, 2H), 7.60 – 7.57 (m, 1H), 7.56 – 7.54 (m, 2H), 7.48 (*app* t, *J* = 7.7 Hz, 1H), 7.40 – 7.35 (m, 2H), 7.30 – 7.25 (m, 2H), 7.22 (d, *J* = 2.6 Hz, 2H); ^{13}C NMR (100 MHz, $CDCl_3$): δ 157.4, 149.8, 139.9, 138.0, 137.4, 137.0, 129.2, 129.2, 128.8, 128.6, 127.8, 127.2, 126.7, 126.2, 125.2, 122.4, 120.8; HRMS (APCI+): calculated for $C_{19}H_{16}N$ (M + H^+), theoretical 258.1289, measured 258.1275; m.p. 98 – 100 ºC.

2-(3-bromophenyl)piperidine, ***5***

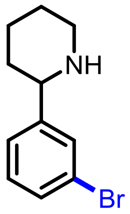

A solution of 0.1M SmI_2 in THF (15 ml, 1.5 mmol, 6.0 eq) was added to 2-(3-bromophenyl)pyridine (58.5 mg, 0.25 mmol, 1.0 eq) under argon. To this, degassed water was added (0.25 ml, 56 mmol, 14 eq) and the reaction was stirred at room temperature for 45 minutes until the colour changed from blue to yellow. 10% aqueous HCl was added to the reaction and it was further stirred for 10 mins. The reaction mixture was then extracted three times with diethyl ether before the aqueous layer was then basified with 10% aqueous NaOH. This was saturated with NaCl and then extracted three times with diethyl ether. After drying the second set of combined organic layers over $MgSO_4$ and concentration *in vacuo*, the crude product was purified by preparative TLC (90% EtOAc: 10% MeOH). The compound was collected as a pale yellow oil (31.2 mg, 52%).

^{1}H NMR (400 MHz, $CDCl_3$): δ 7.54 (*app* t, *J* = 1.8 Hz, 1H), 7.36 (m, 1H), 7.28 (m, 1H), 7.17 (*app* t, *J* = 7.8 Hz, 1H), 3.58 – 3.54 (m, 1H), 3.18 (*app* dt, *J* = 11.7, 2.0 Hz, 1H), 2.77 (*app* td, *J* = 11.7, 2.9 Hz, 1H), 2.12 (br s, 1H), 1.89 (m, 1H), 1.77 (m, 1H), 1.65 (ddd, *J* = 12.0, 4.3, 2.1 Hz, 1H), 1.59 – 1.43 (m, 3H); ^{13}C NMR (100 MHz, $CDCl_3$): δ 147.8, 130.3 (CH), 130.1 (CH), 129.9 (CH), 125.5 (CH), 122.6, 61.9 (CH), 47.7 (CH_2), 35.0 (CH_2), 25.8 (CH_2), 25.4 (CH_2); HRMS (EI): calculated for $C_{11}H_{14}NBr$, theoretical 239.0304, measured 239.0294.

References

1) M. Szostak, M. Spain, D. J. Procter *Nat. Protocol* **2012**, *7,* 970-977
2) A. M. Clark, C. E. F. Rickard, W. R. Roper, L. J. Wright *Organometallics* **1999,** *18*, 2813–2820
3) A. S. Ionkin , W. J. Marshall, B. M. Fish *Organometallics*, **2006**, *25*, 1461–1471
4) I. Sasaki, L. Vendier, A. Sournia-Saquet, P. G. Lacroix *Eur. J. Inorg. Chem.* **2006**, 3294–3302
5) D. F. Crépin, J. P. Harrity *Org. Lett.*, **2013**, *15*, 4222–4225.

2-(3-bromophenyl)pyridine

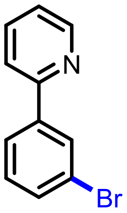

1H NMR, 400 MHz, CDCl3

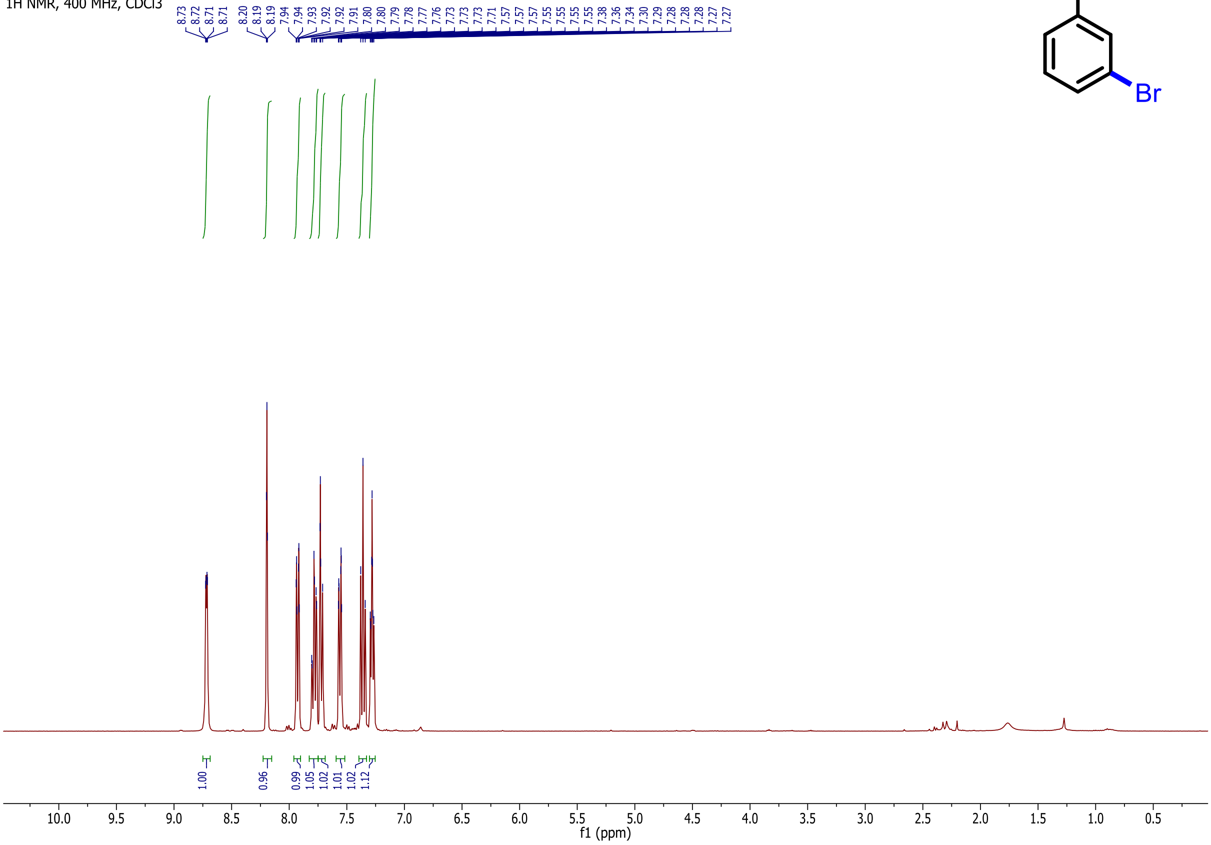

13C NMR, 100 MHz, CDCl3

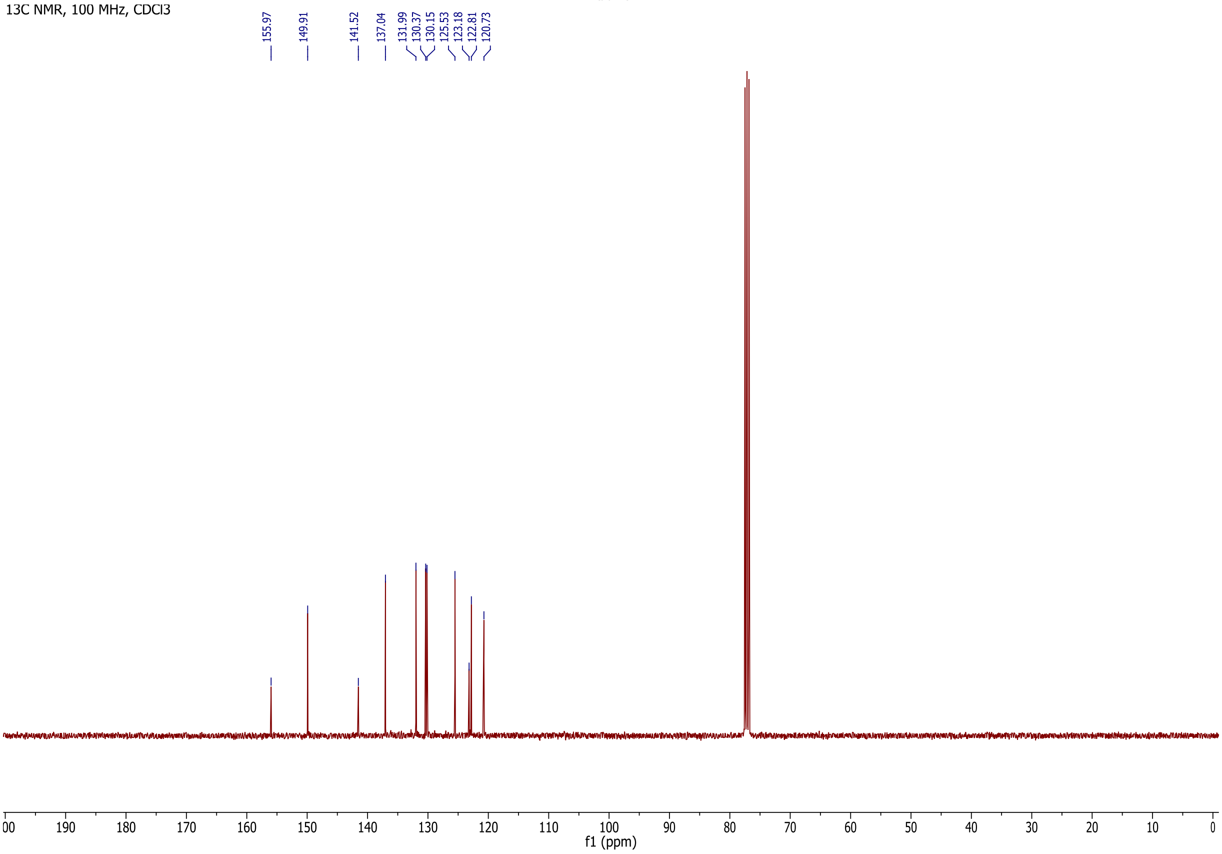

2-(3-bromo-4-methylphenyl)pyridine

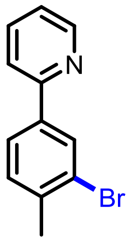

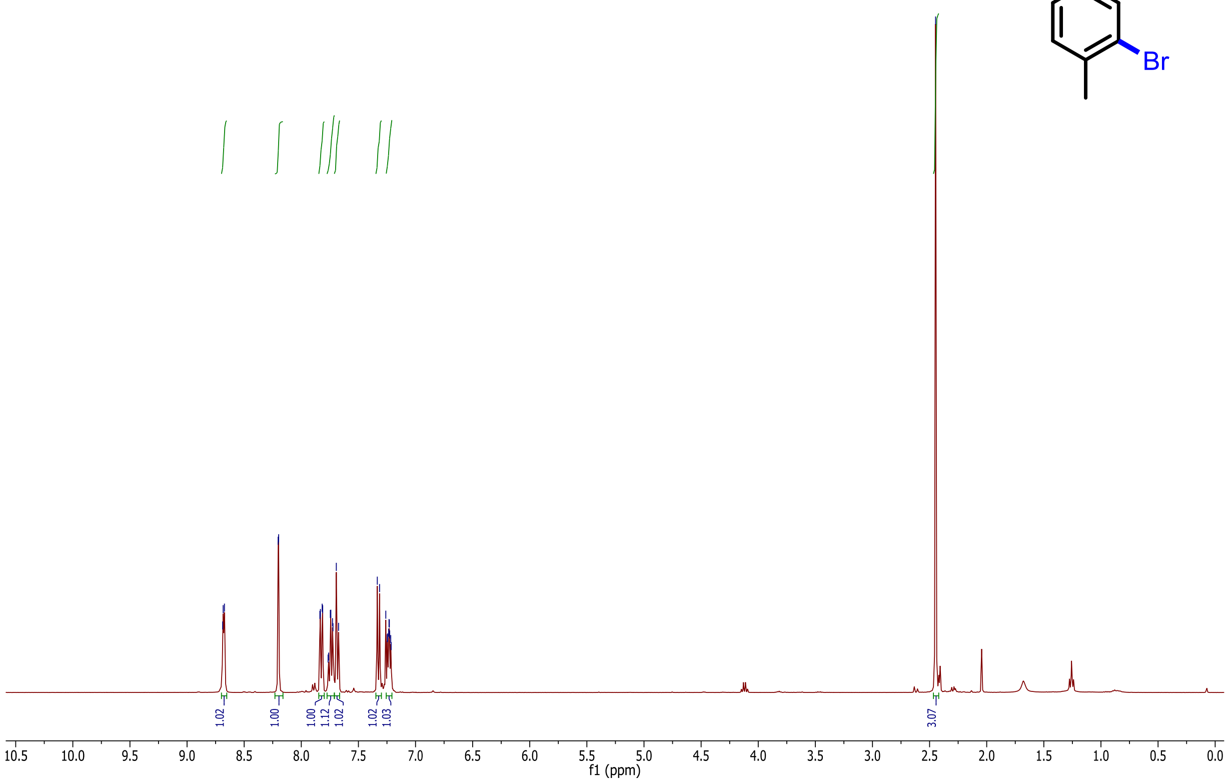

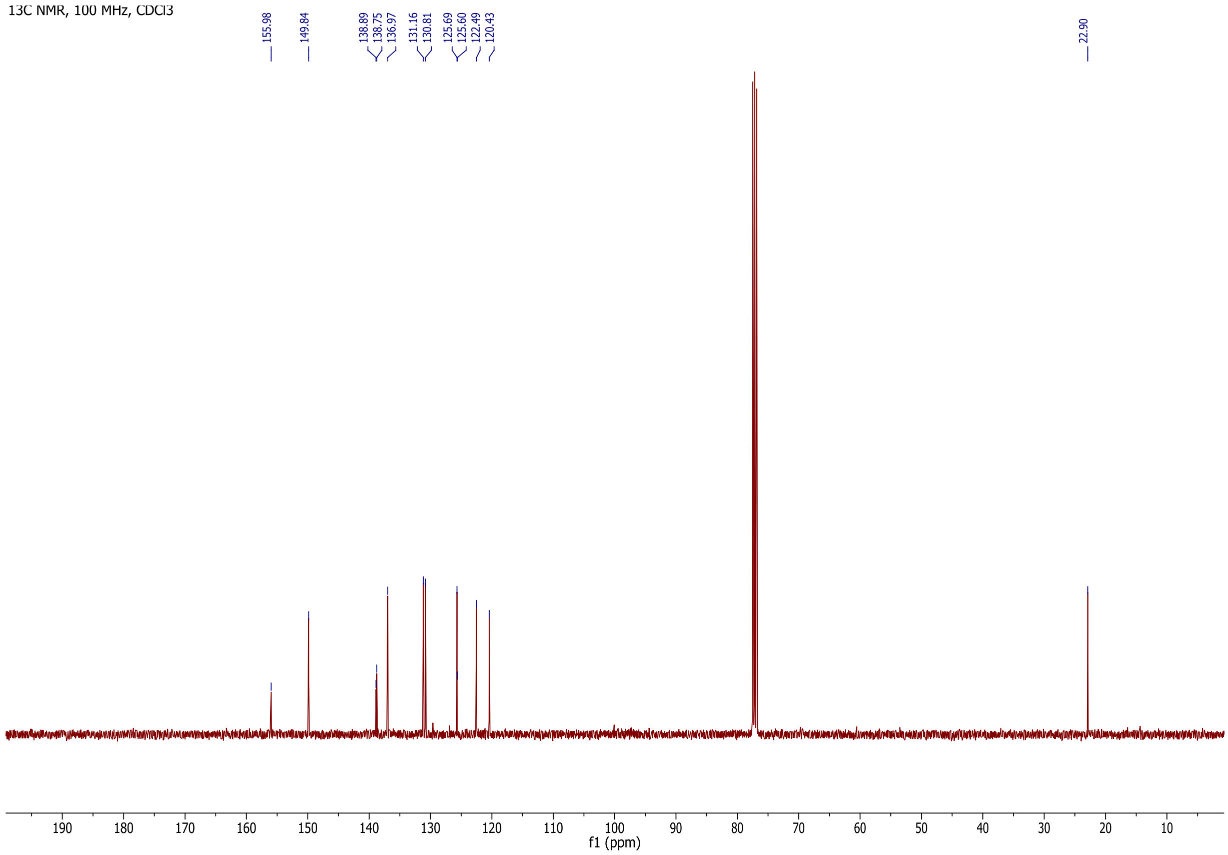

N-(2-bromo-4-(pyridin-2-yl)phenyl)acetamide

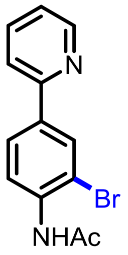

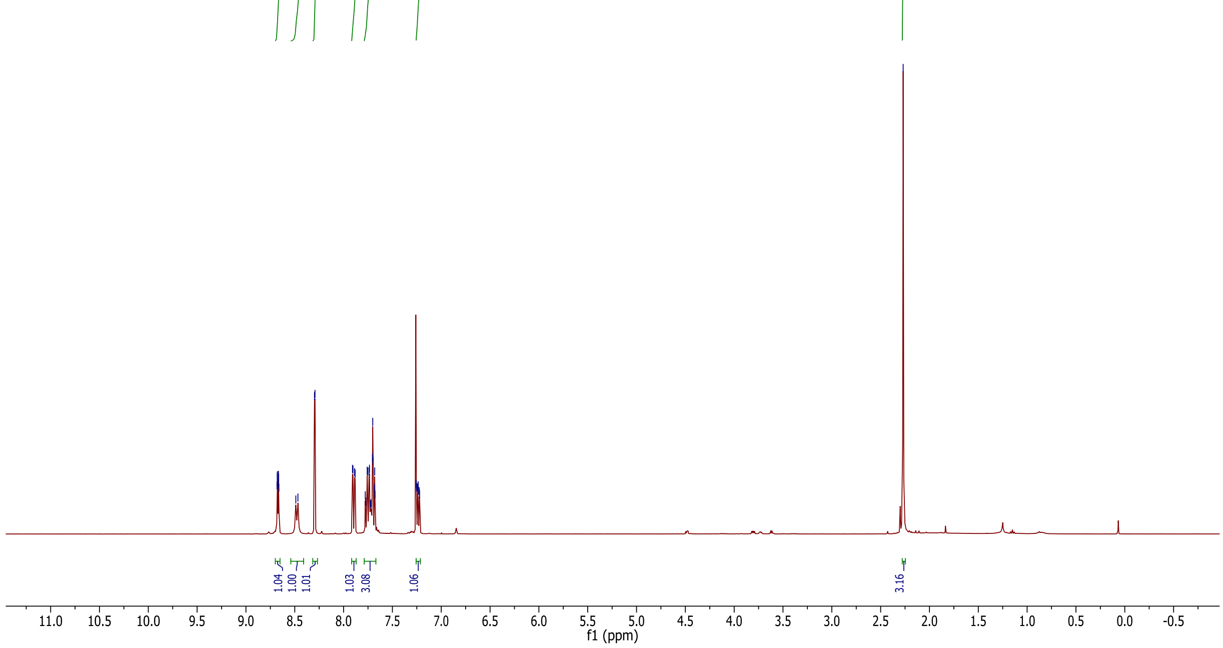

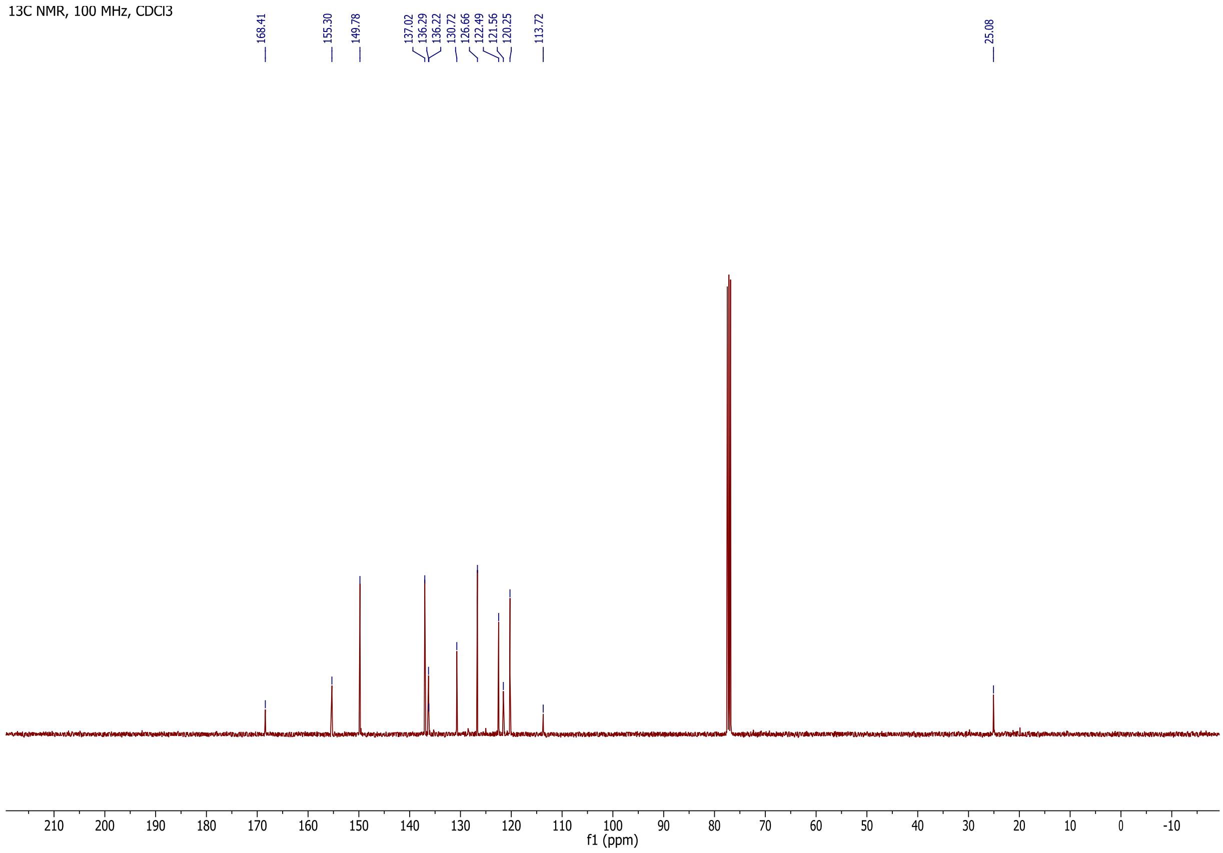

2-(3-bromo-4-(tert-butyl)phenyl)pyridine

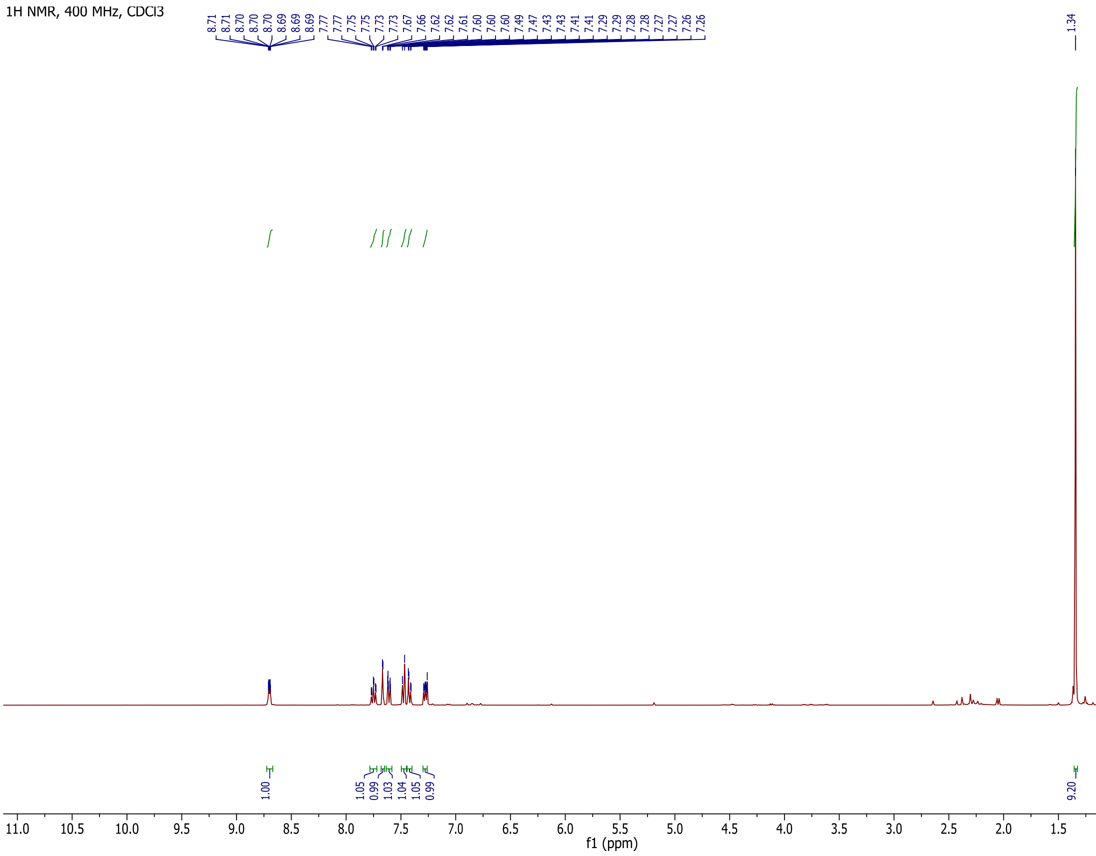

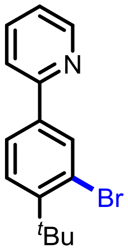

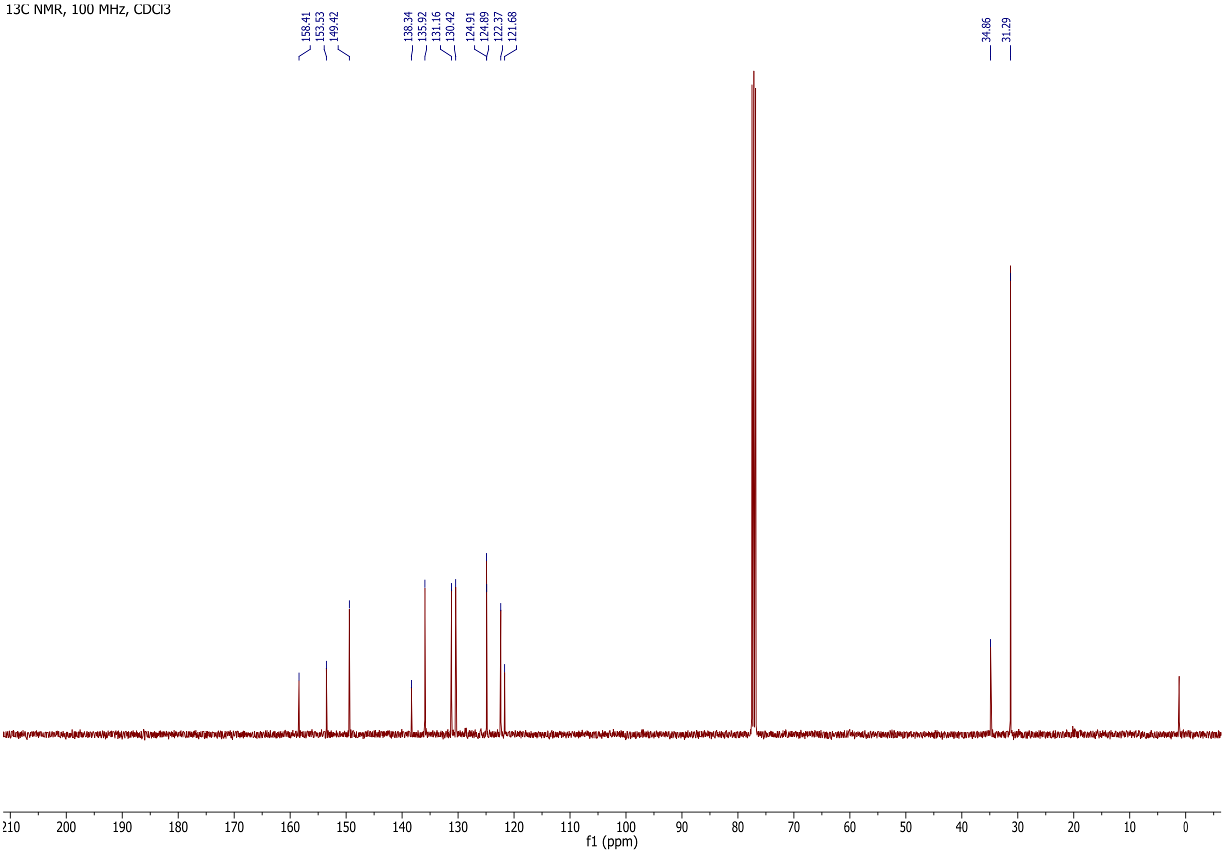

2-(3-bromo-4-fluorophenyl)pyridine

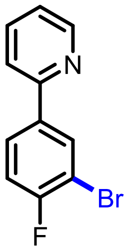

1H NMR, 400 MHz, CDCl3

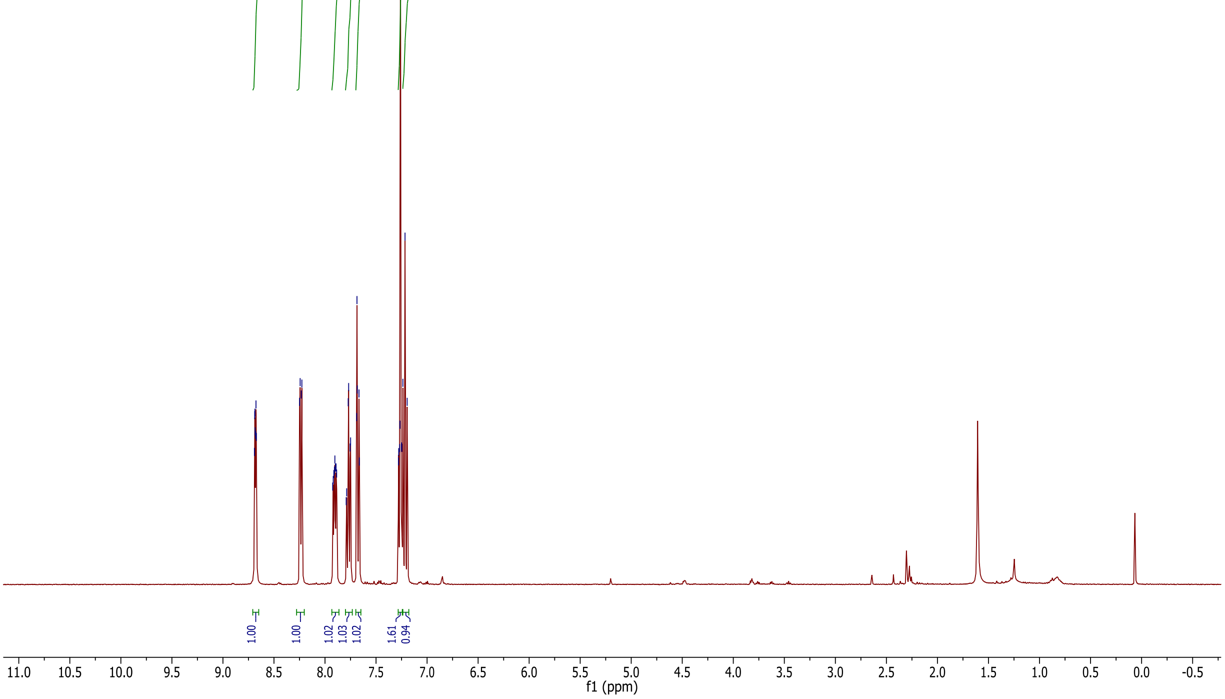

13C NMR, 100 MHz, CDCl3

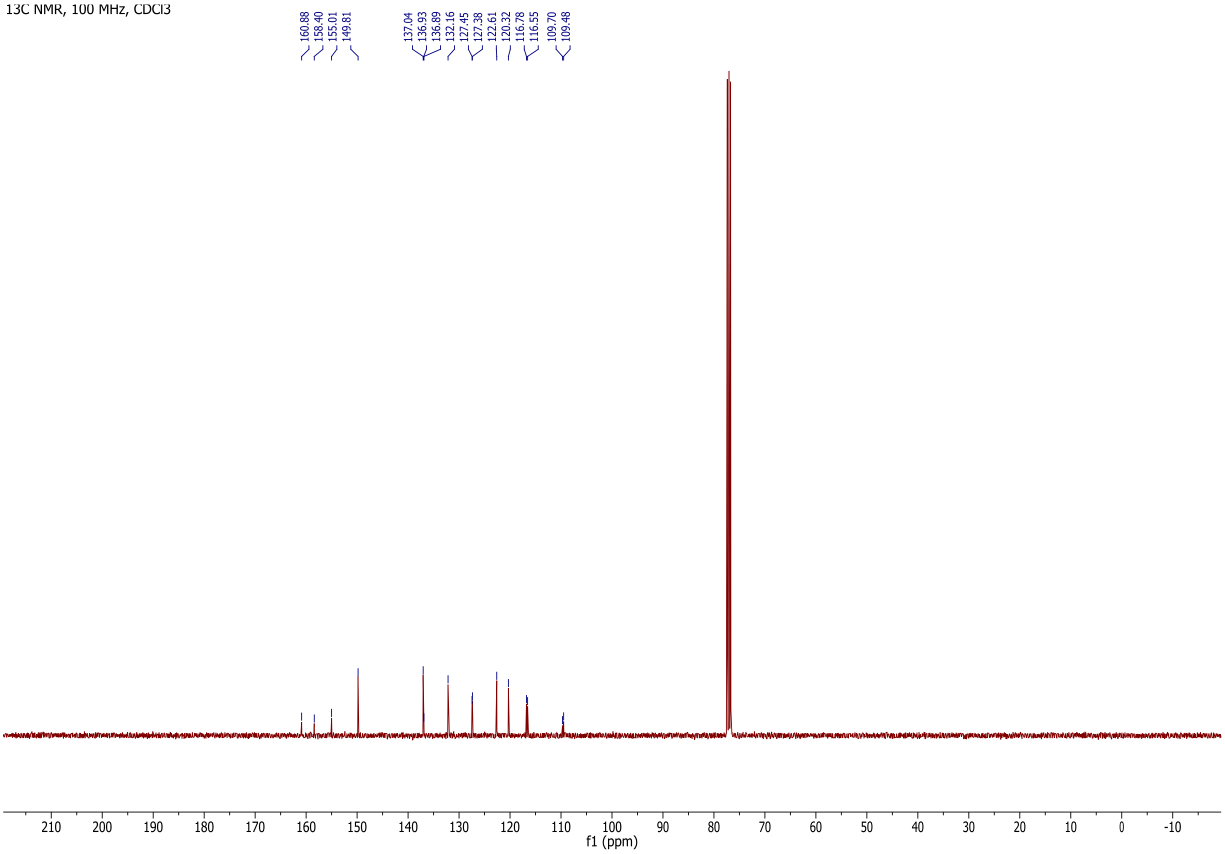

19F NMR, 376 MHz, CDCl3

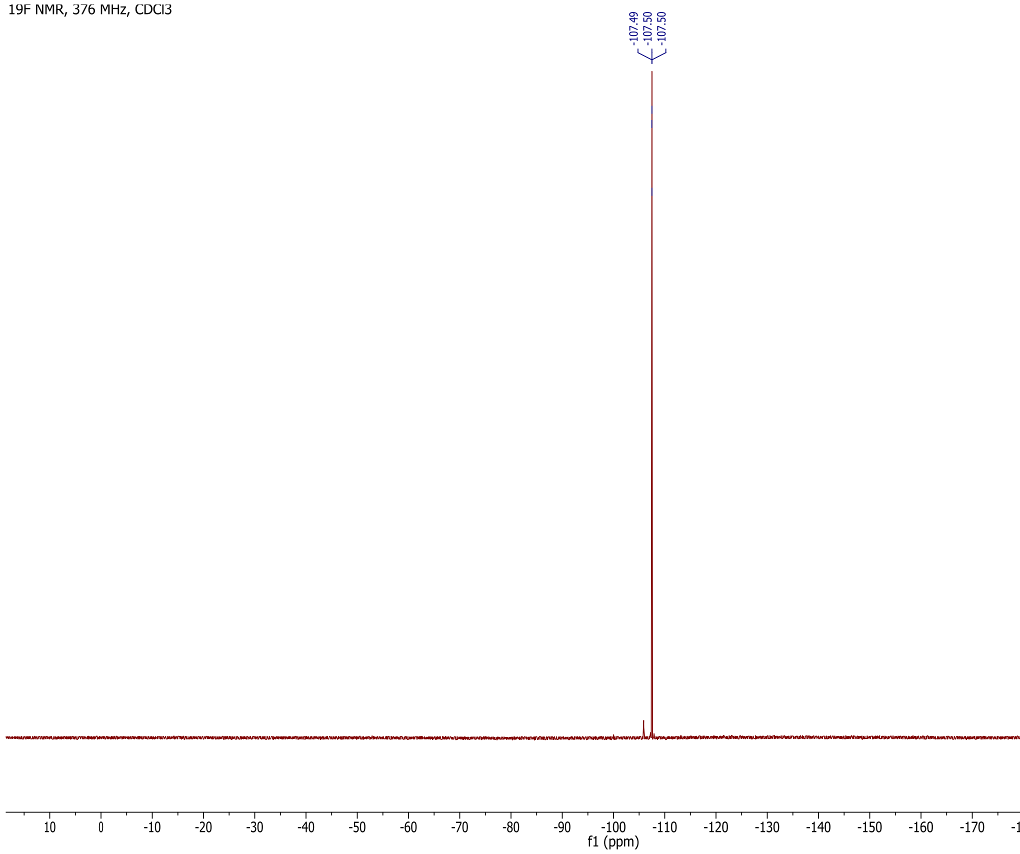

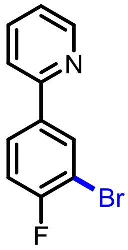

2-bromo-4-(pyridin-2-yl)benzaldehyde

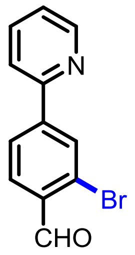

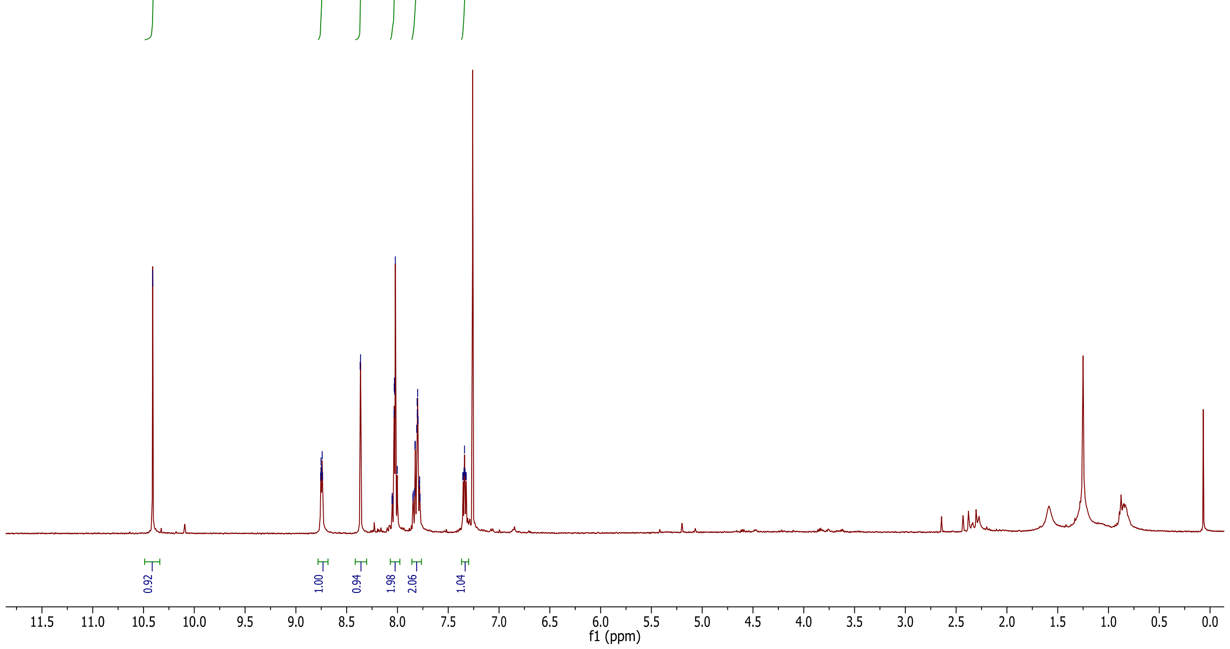

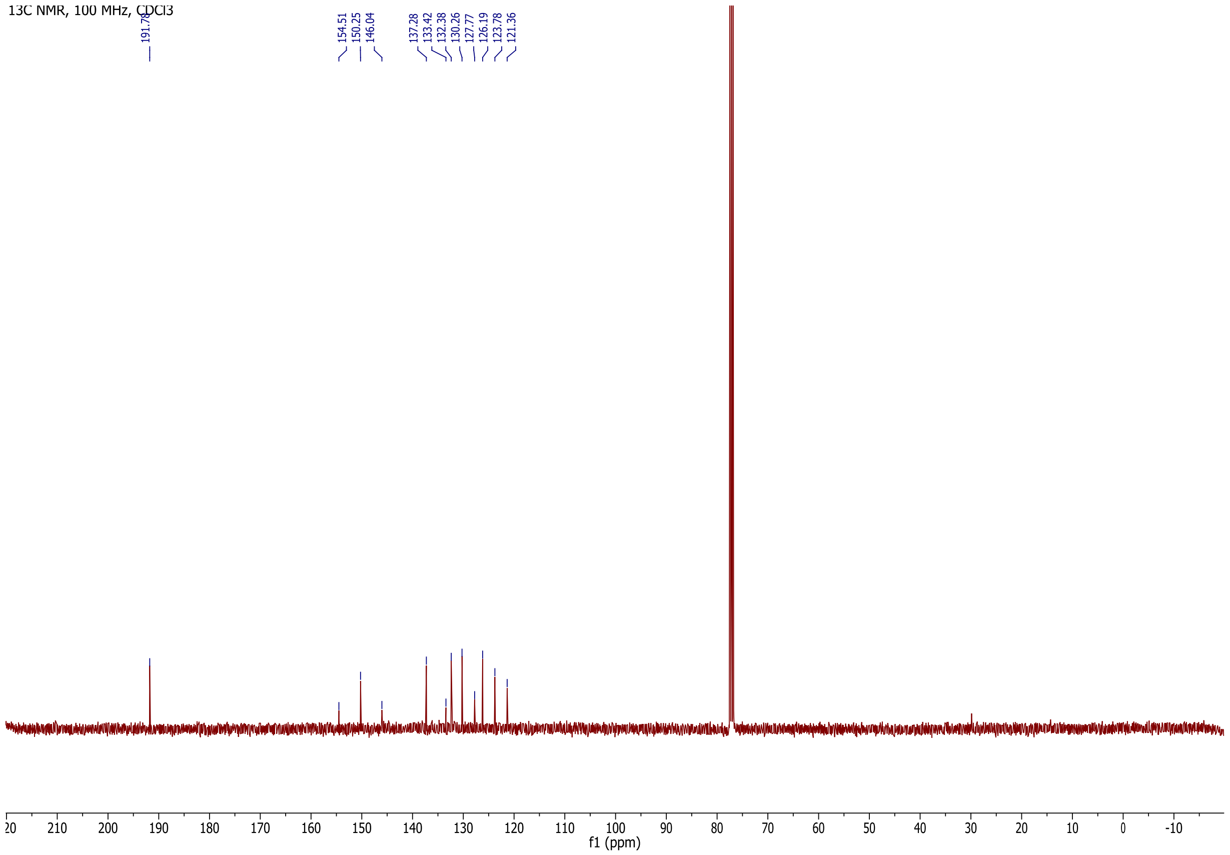

2-(3-bromo-4-chlorophenyl)pyridine

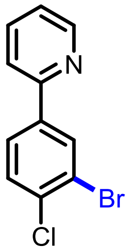

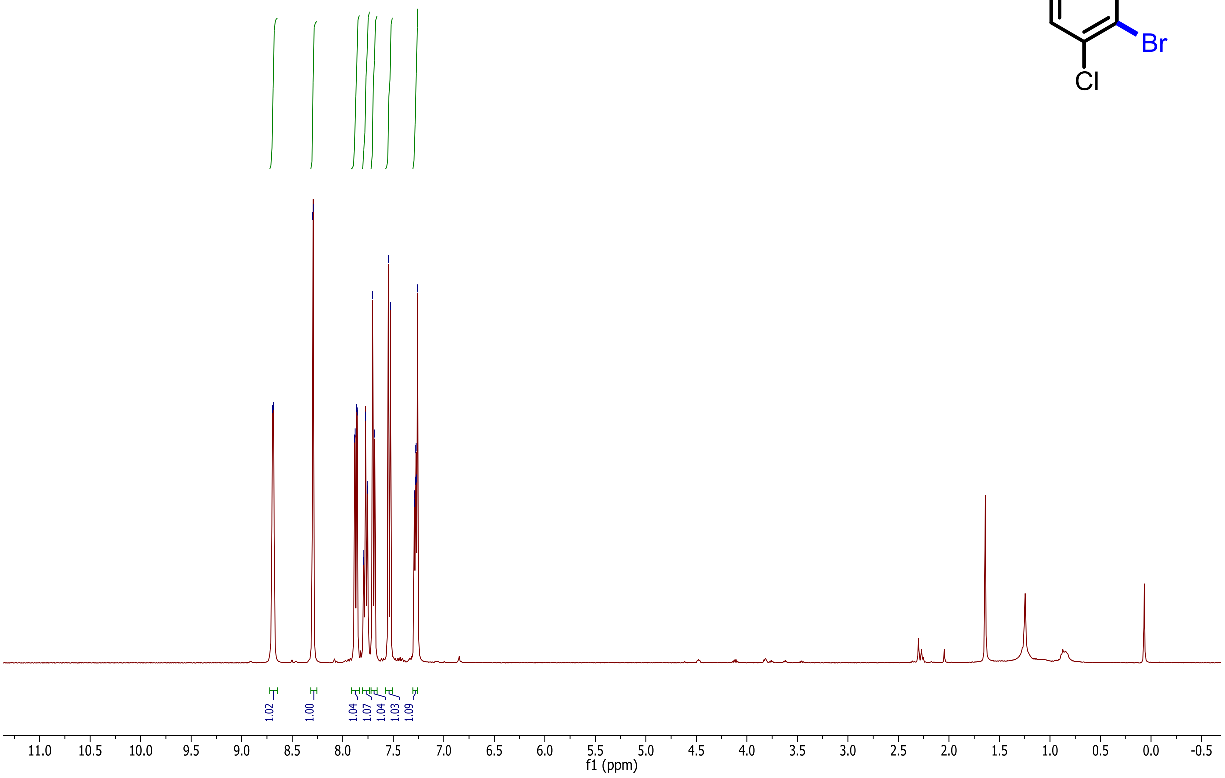

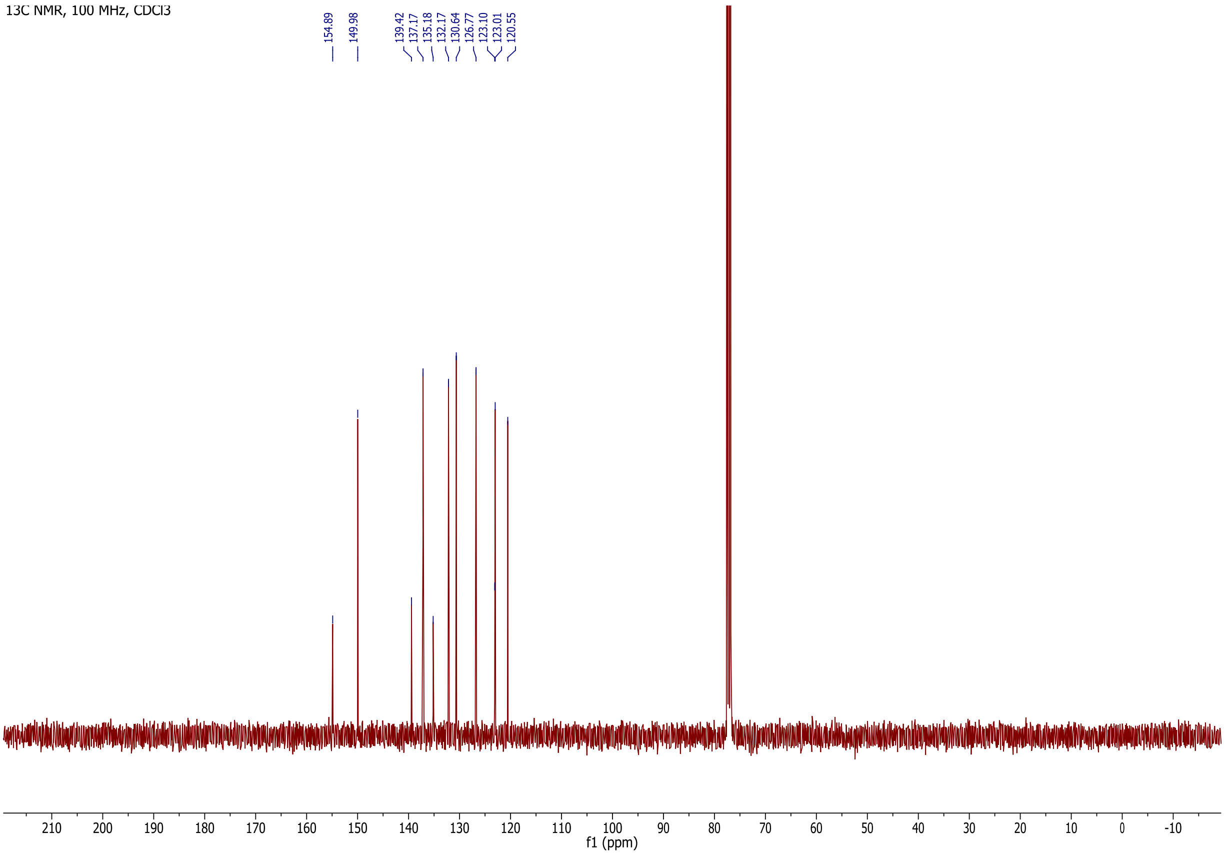

2-(3-bromophenyl)-4-methylpyridine

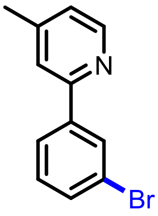

1H NMR, 400 MHz, CDCl3

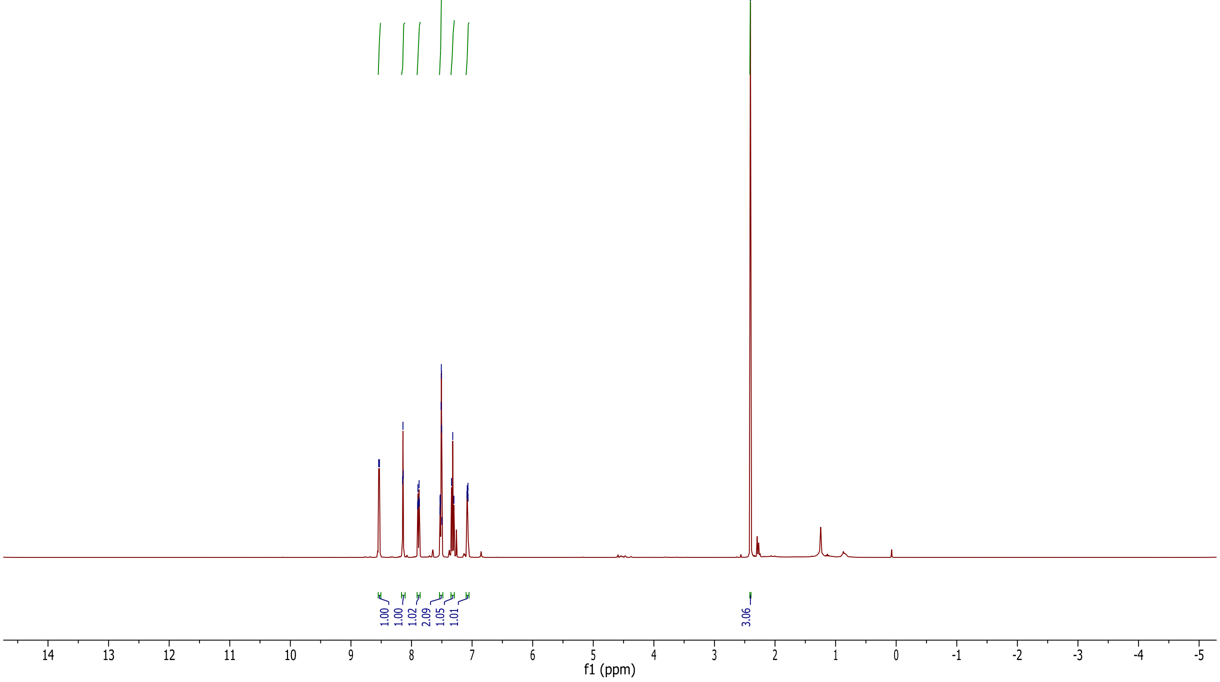

13C NMR, 100 MHz, CDCl3

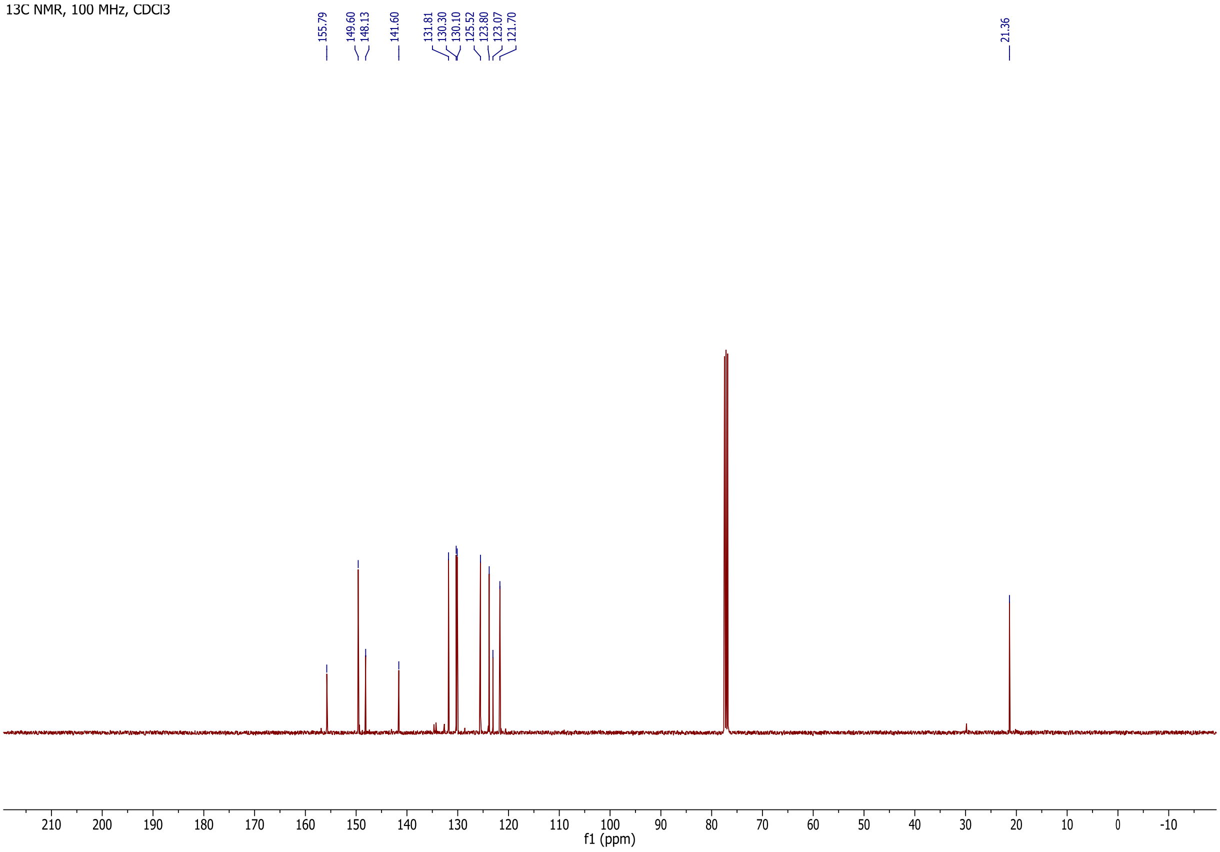

2-(3-bromophenyl)-5-methylpyridine

1H NMR, 400 MHz, CDCl3

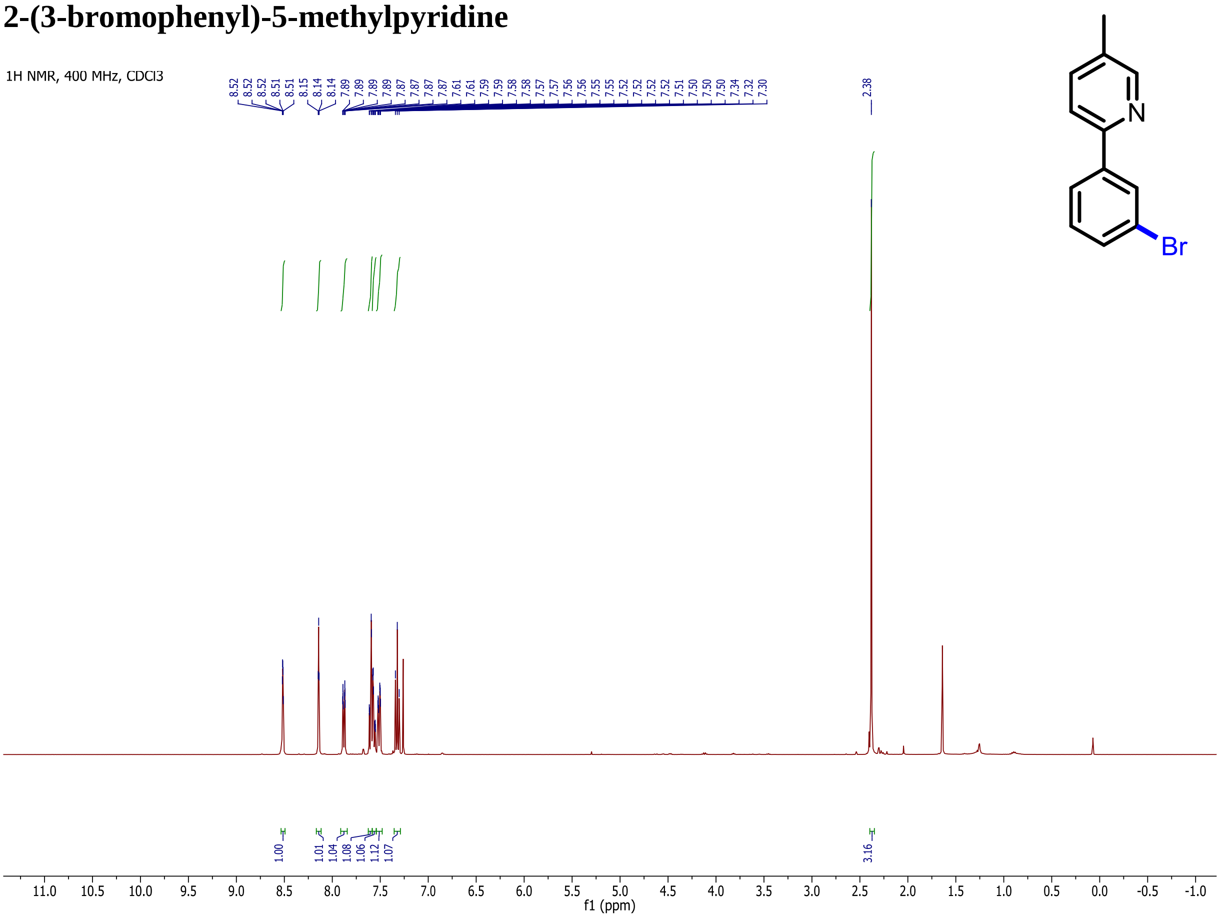

13C NMR, 100 MHz, CDCl3

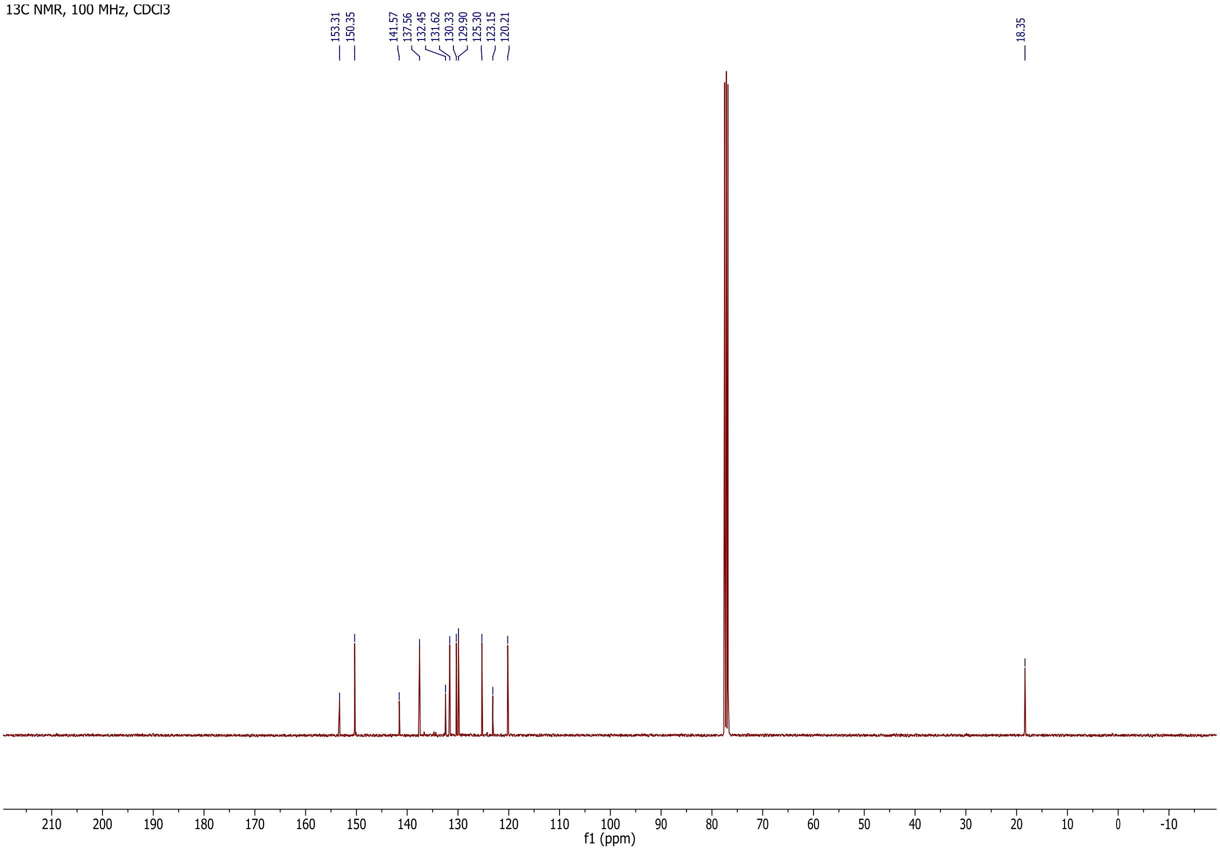

7-bromobenzo[h]quinolone

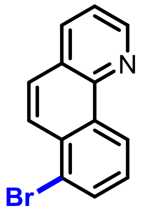

1H NMR, 400 MHz, CDCl3

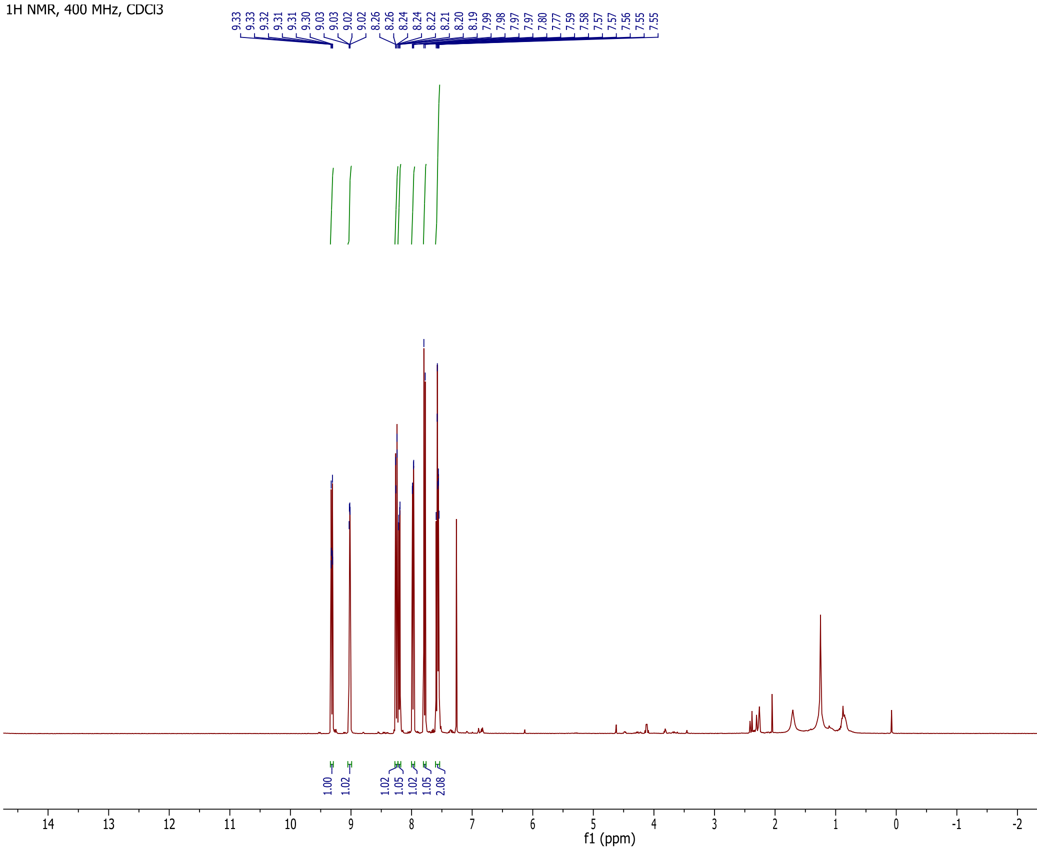

13C NMR, 100 MHz, CDCl3

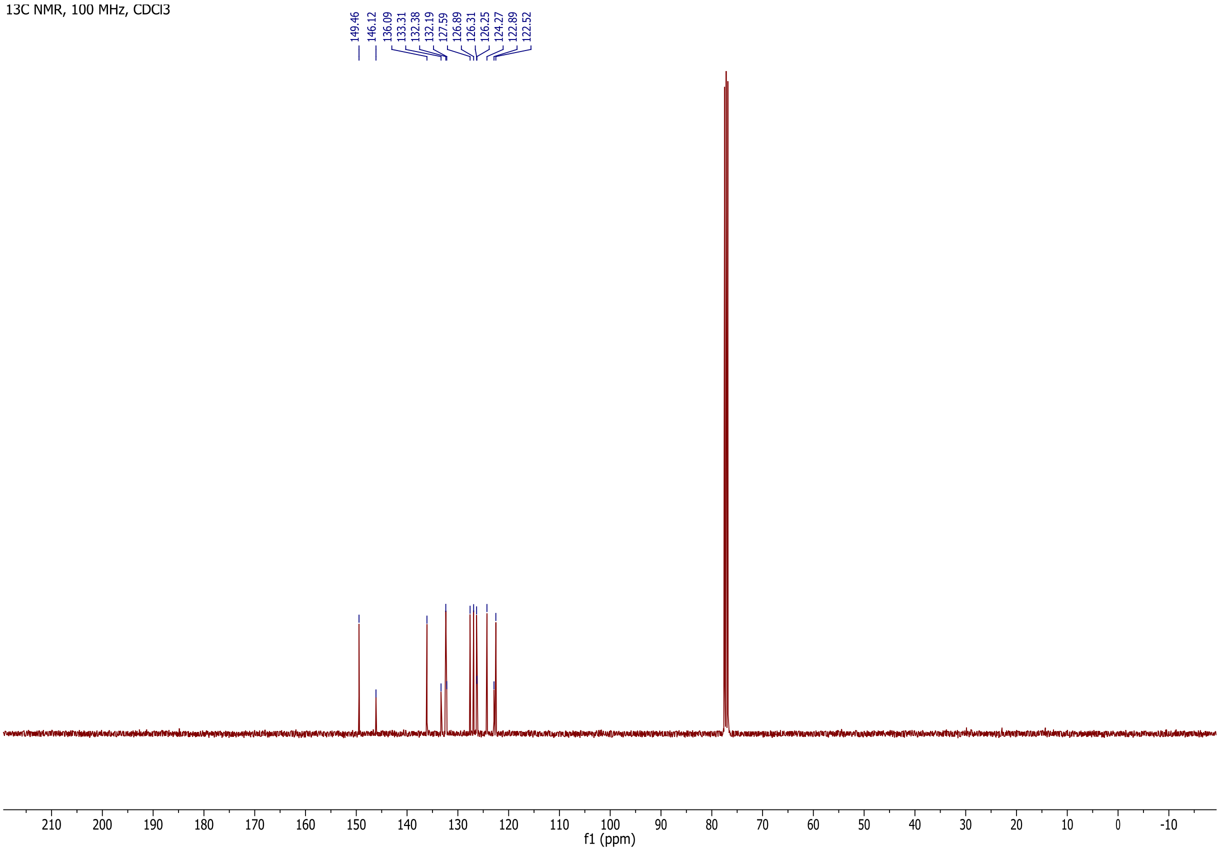

2-(4'-methoxy-[1,1'-biphenyl]-3-yl)pyridine

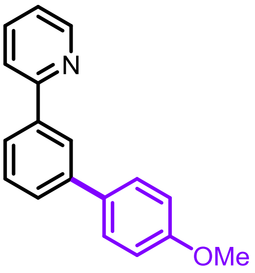

1H NMR, 400 MHz, CDCl3

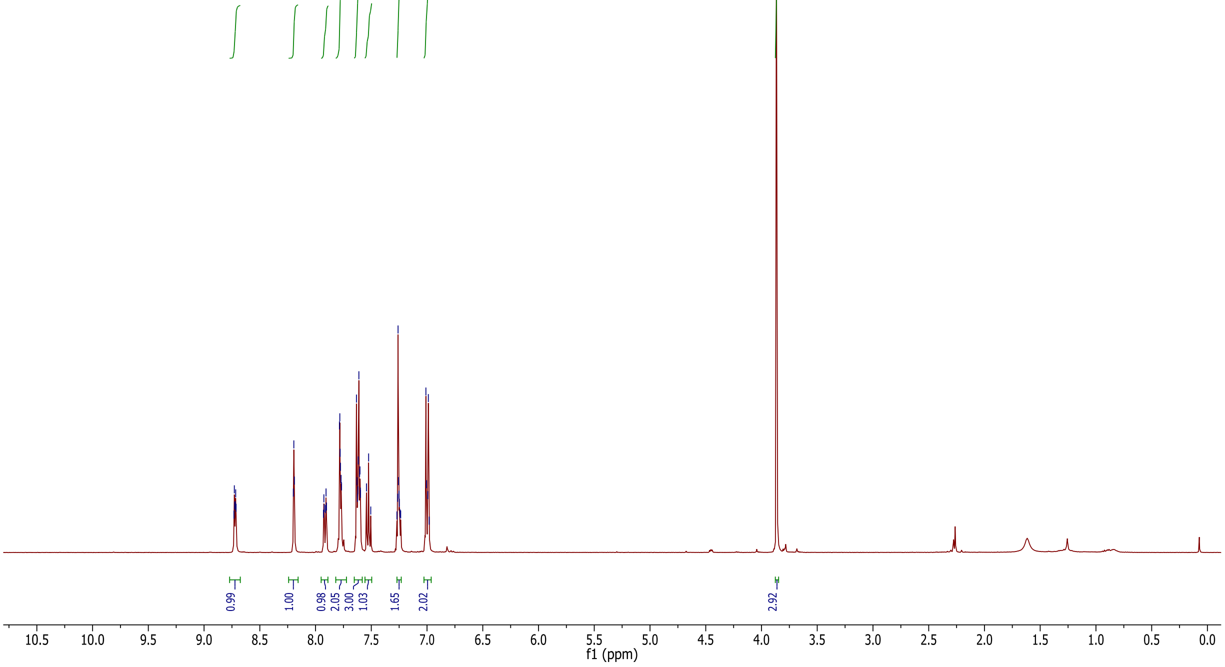

13C NMR, 100 MHz, CDCl3

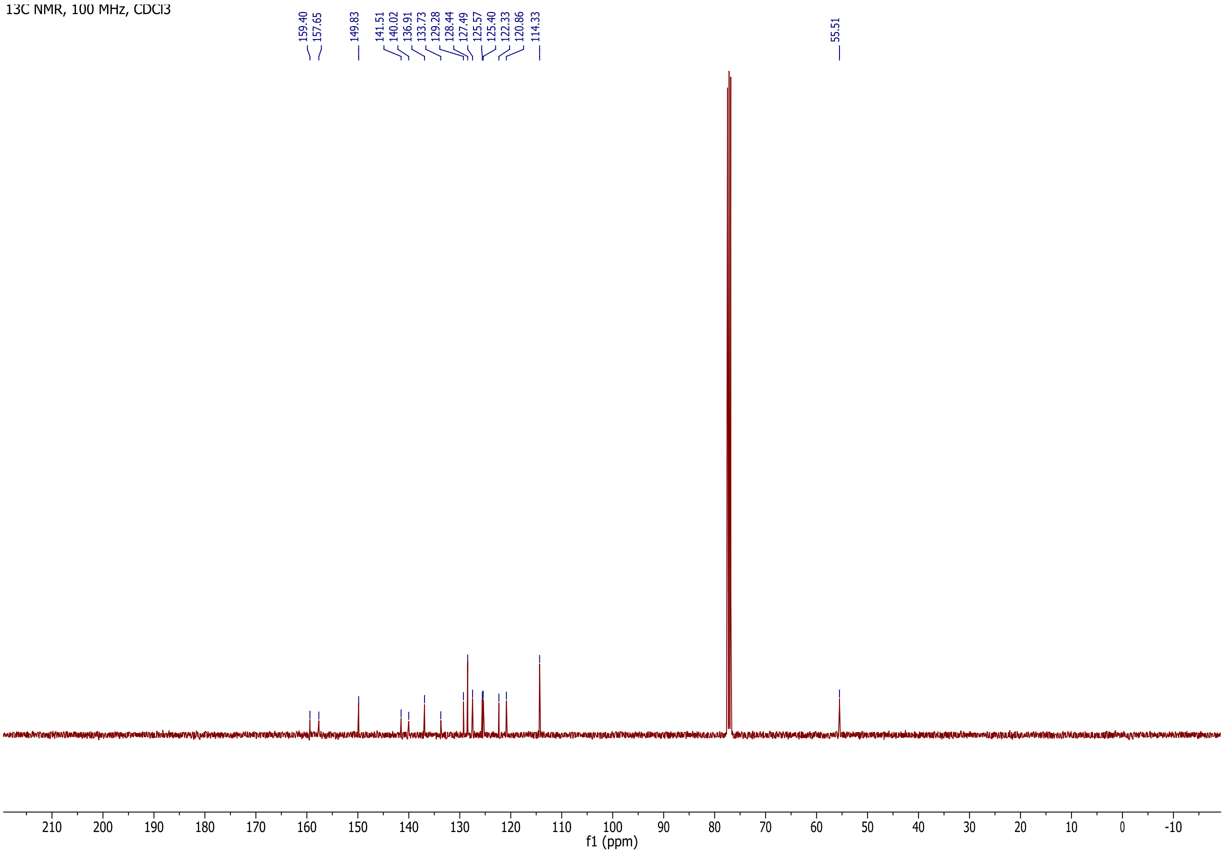

2-(4'-chloro-[1,1'-biphenyl]-3-yl)pyridine

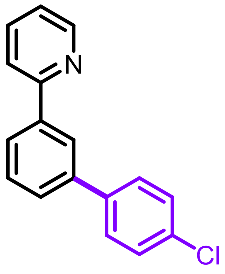

1H NMR, 400 MHz, CDCl3

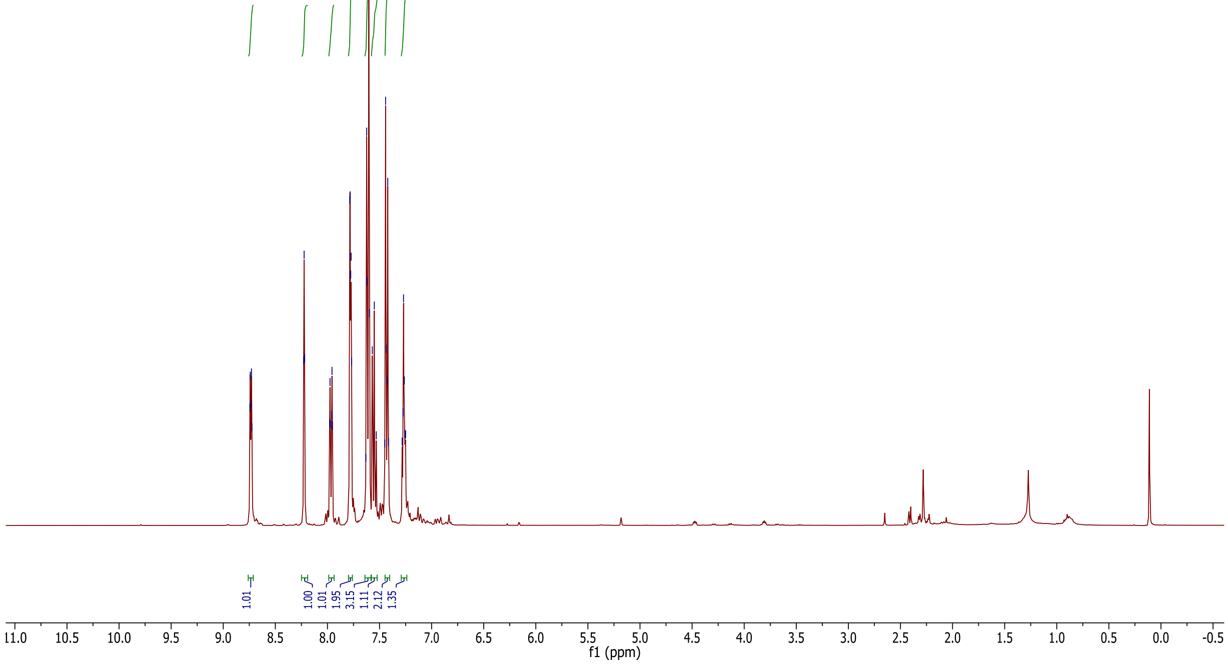

13C NMR, 100 MHz, CDCl3

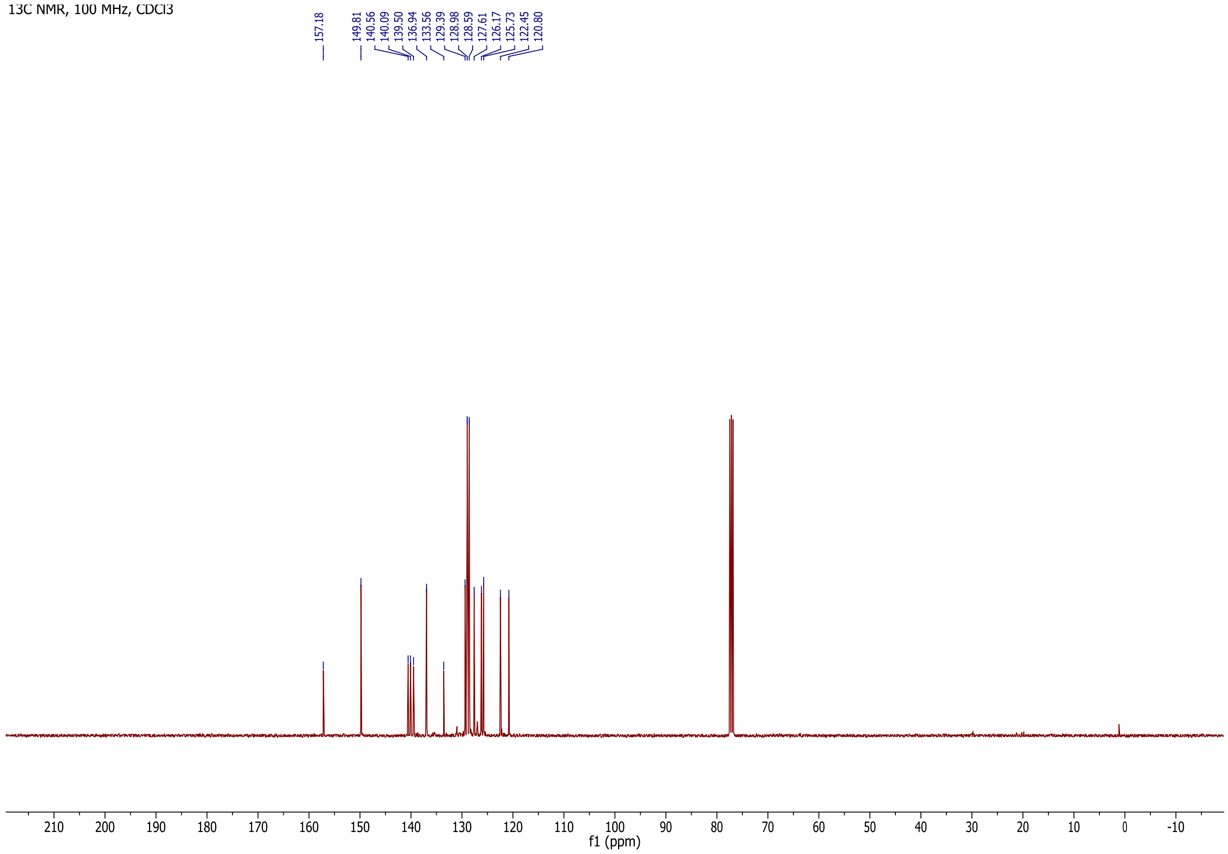

2-([1,1'-biphenyl]-3-yl)pyridine

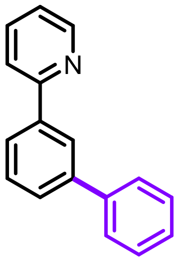

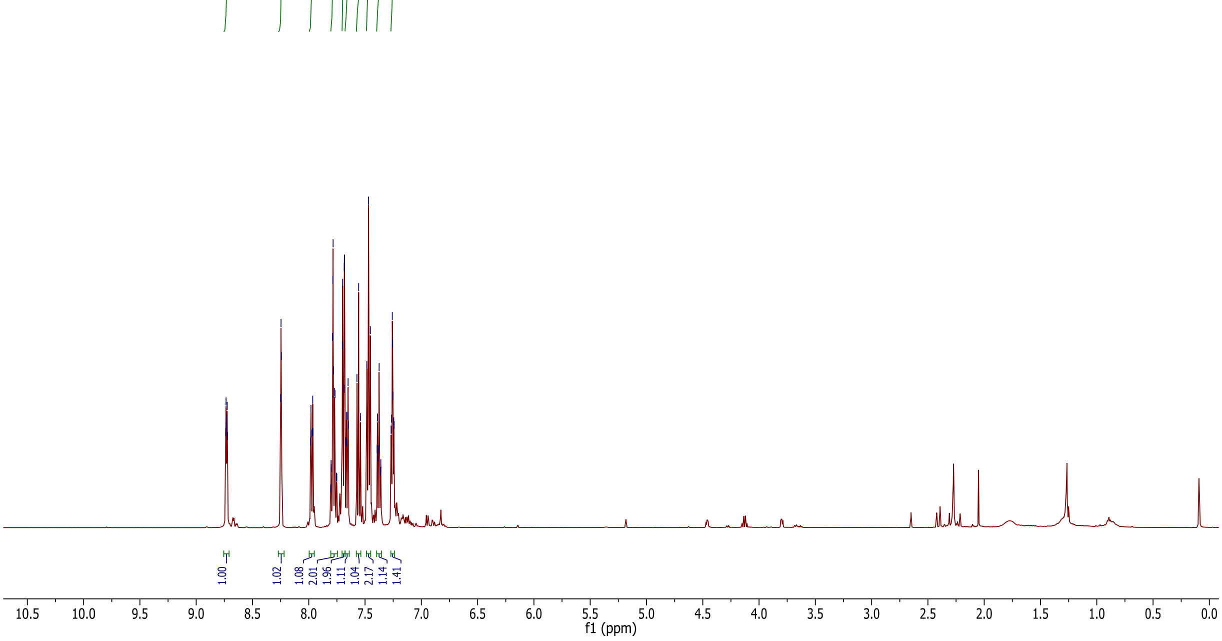

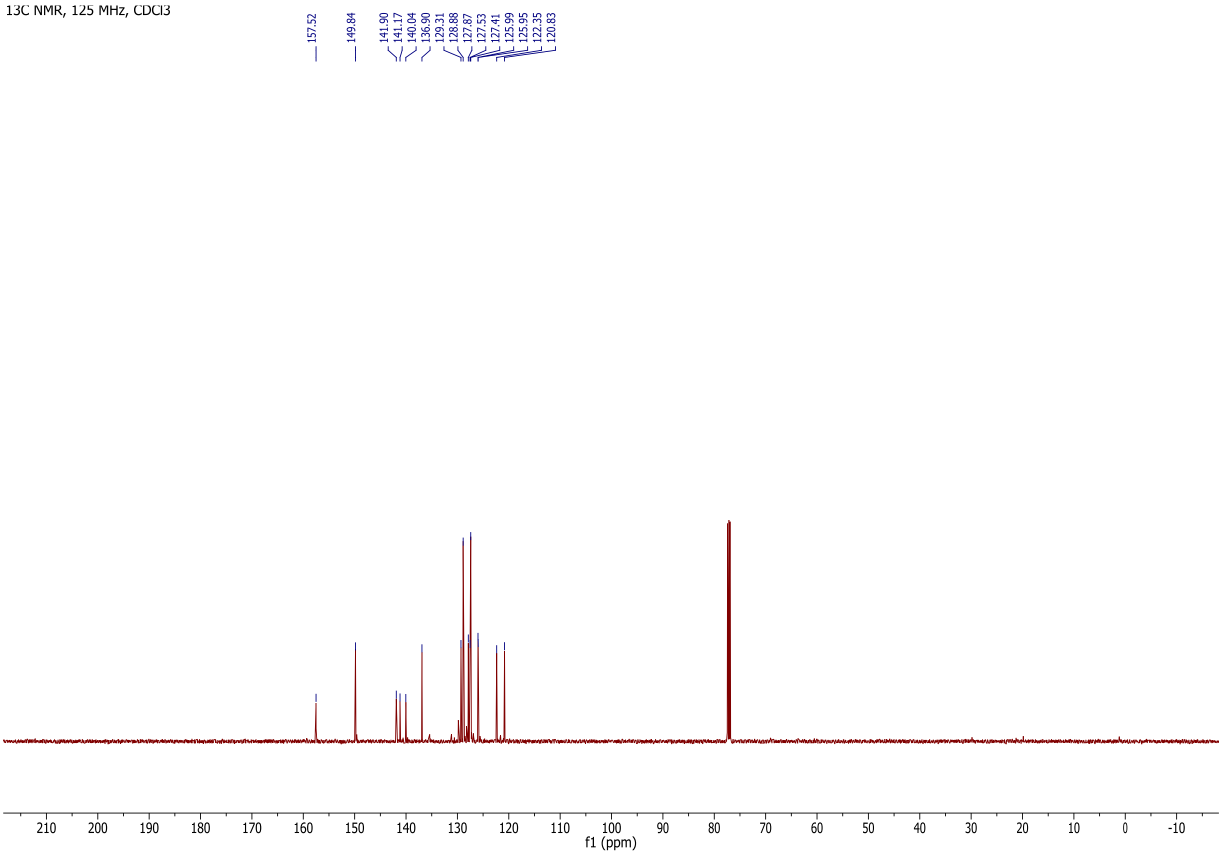

2-(2'-methyl-[1,1'-biphenyl]-3-yl)pyridine

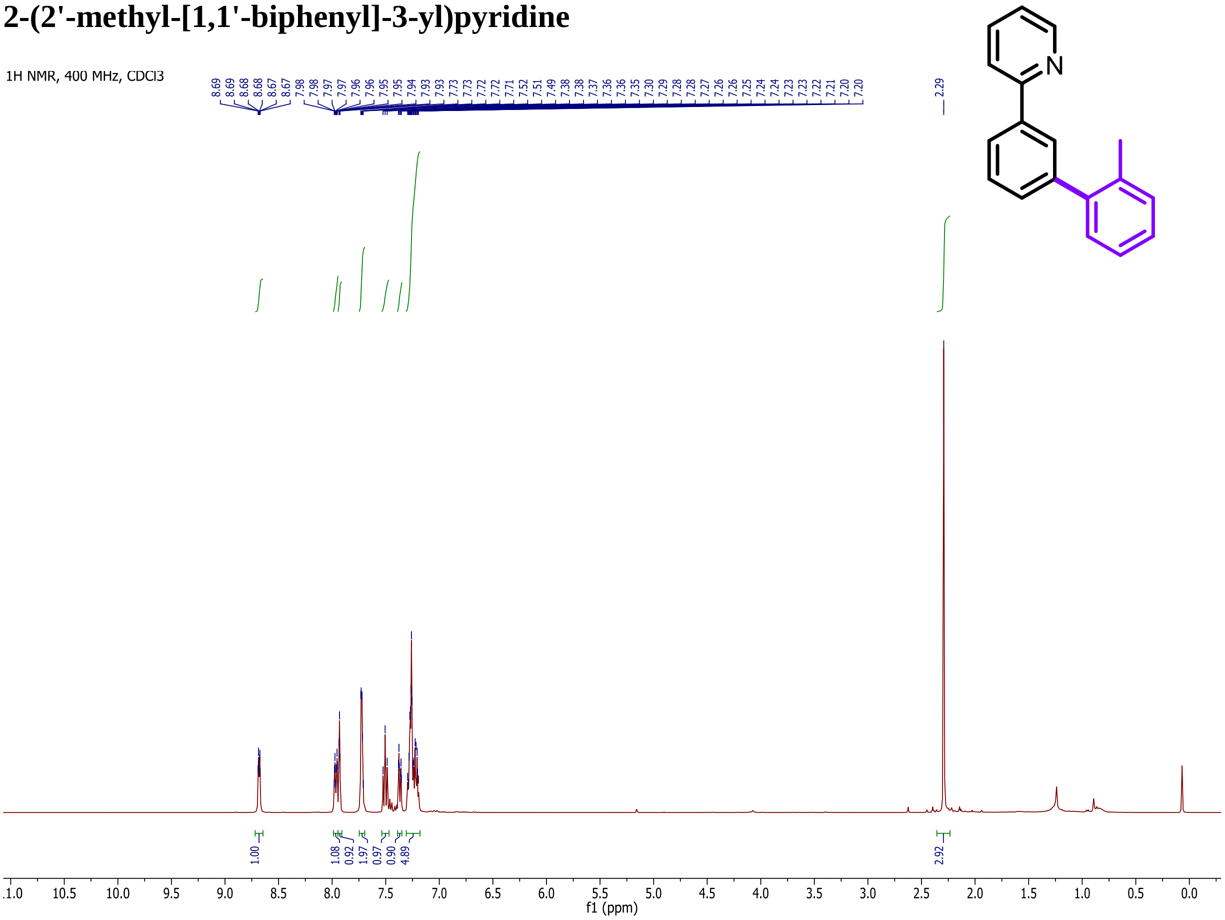

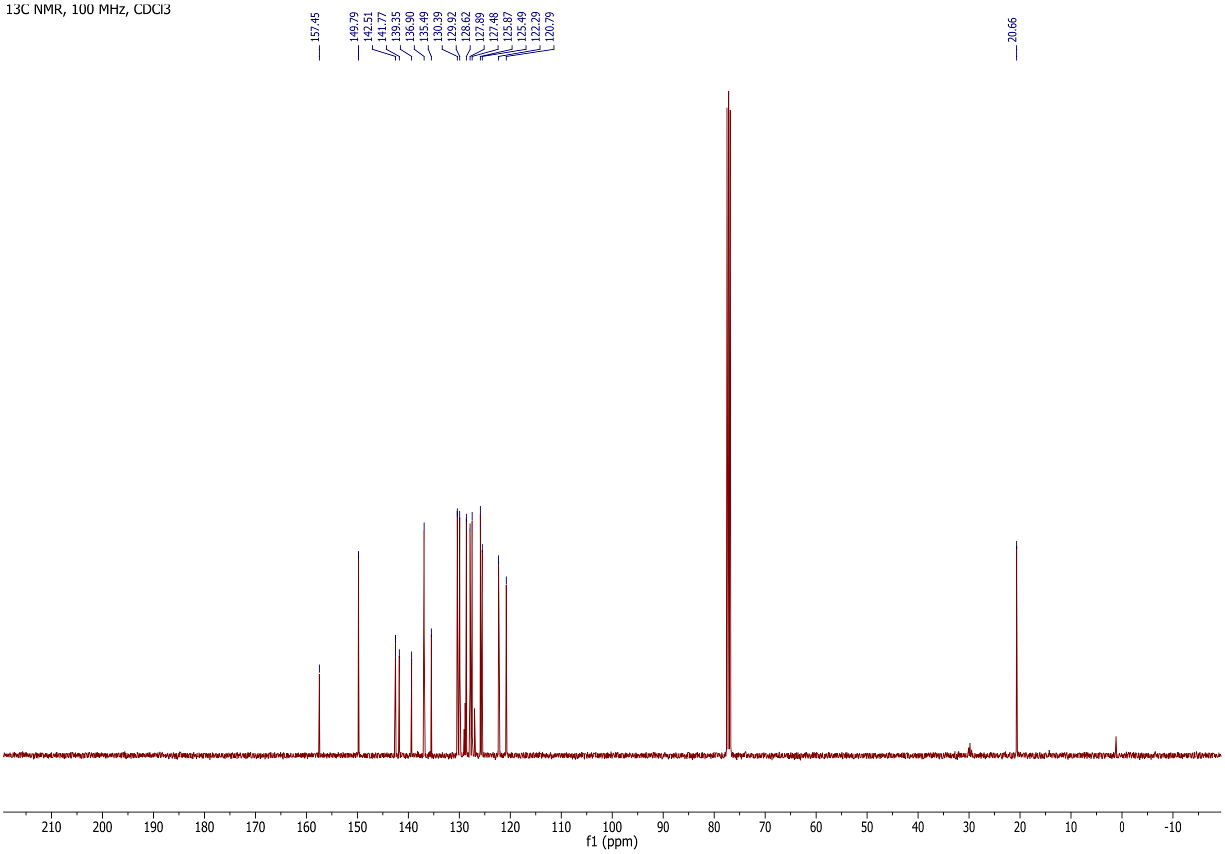

2-(3-(1H-pyrrol-2-yl)phenyl)pyridine

1H NMR, 400 MHz, CDCl3

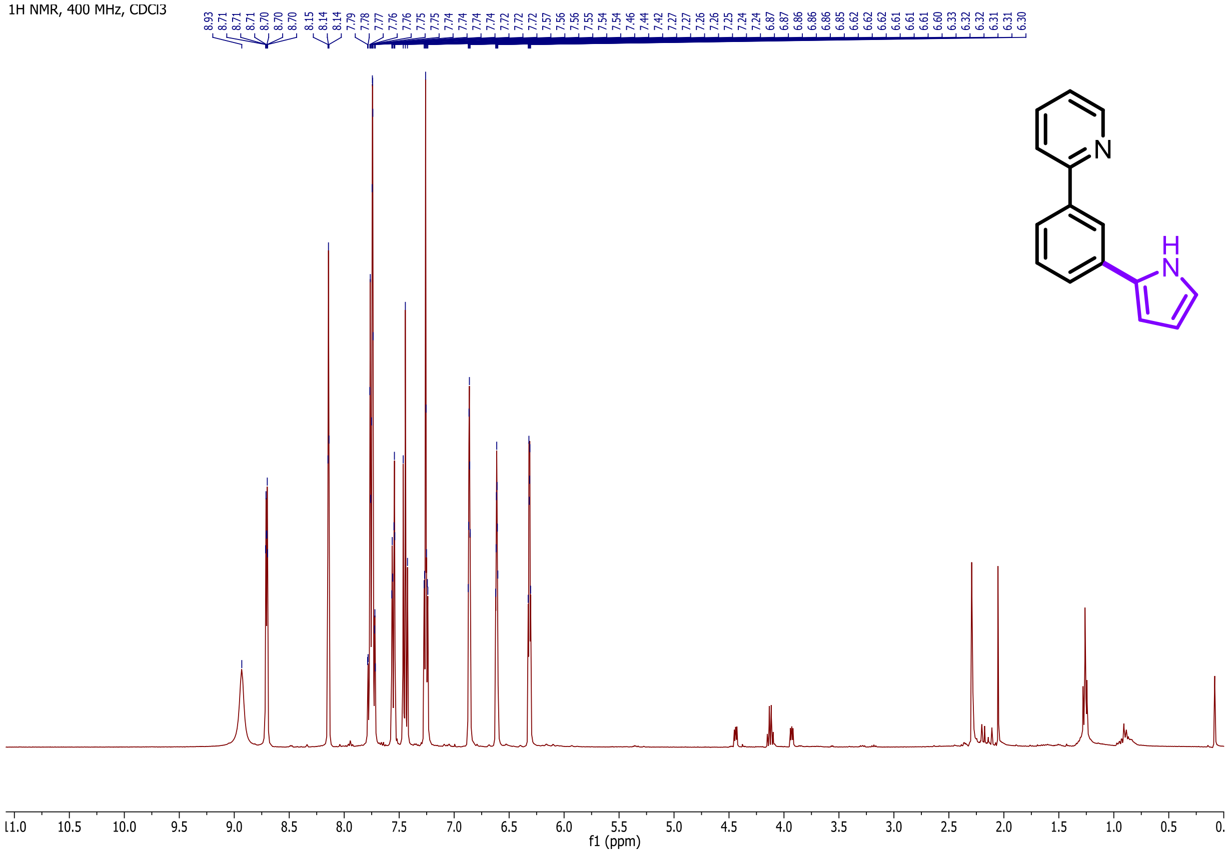

13C NMR, 100 NMR, CDCl3

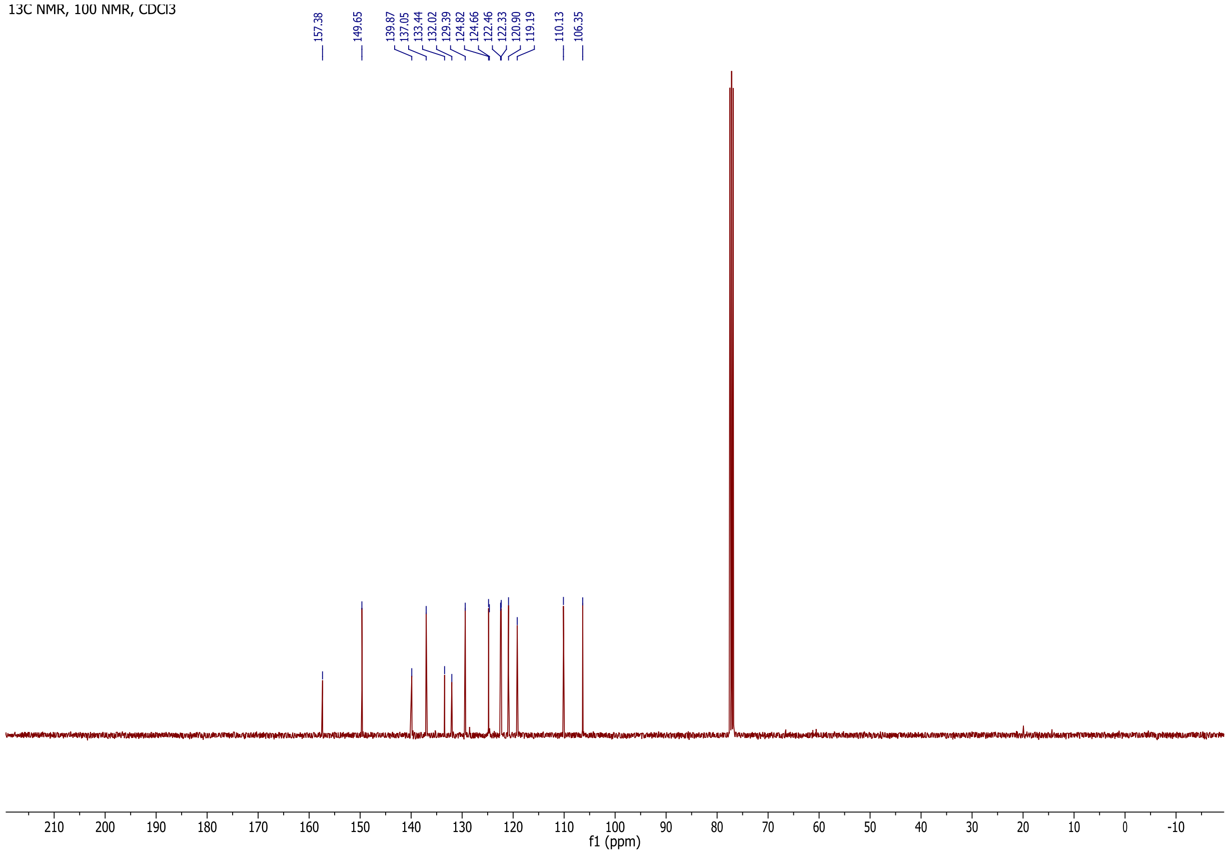

2-(3'-methyl-[1,1'-biphenyl]-3-yl)pyridine

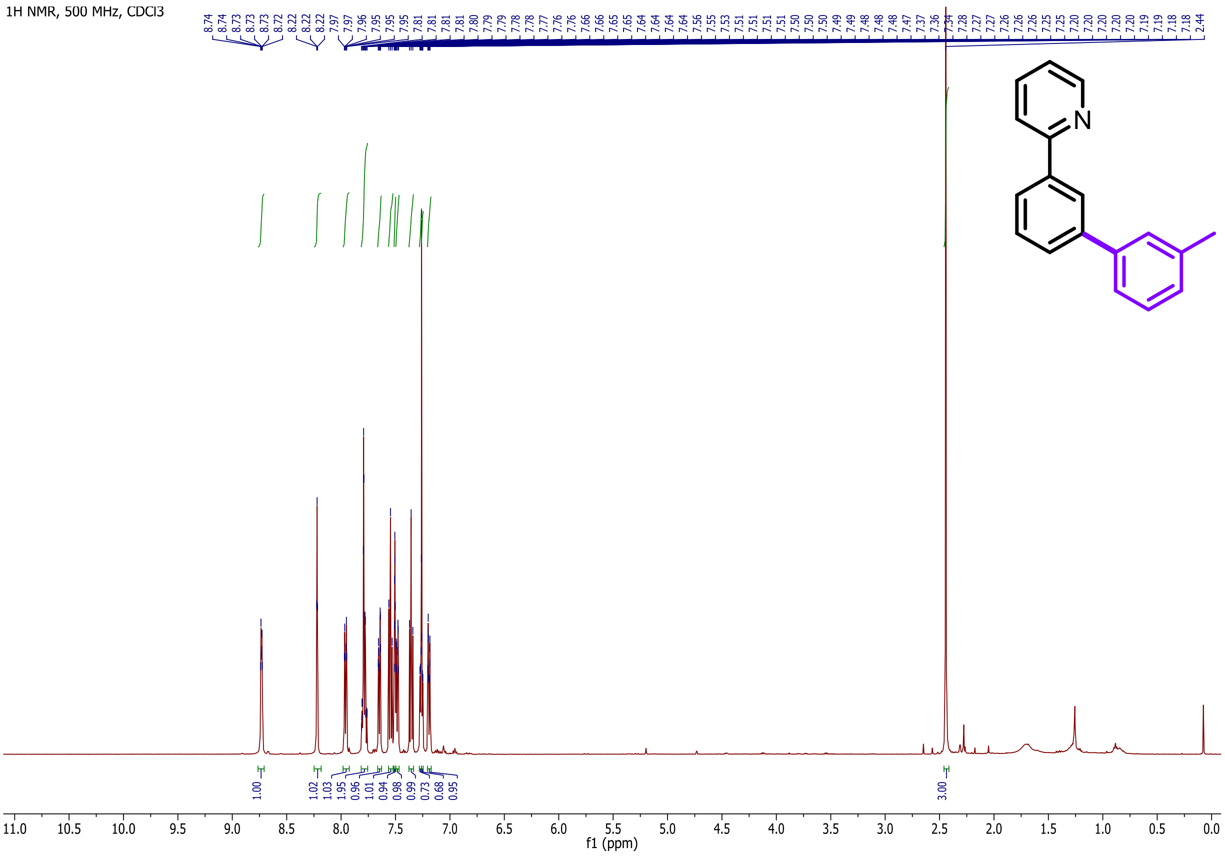

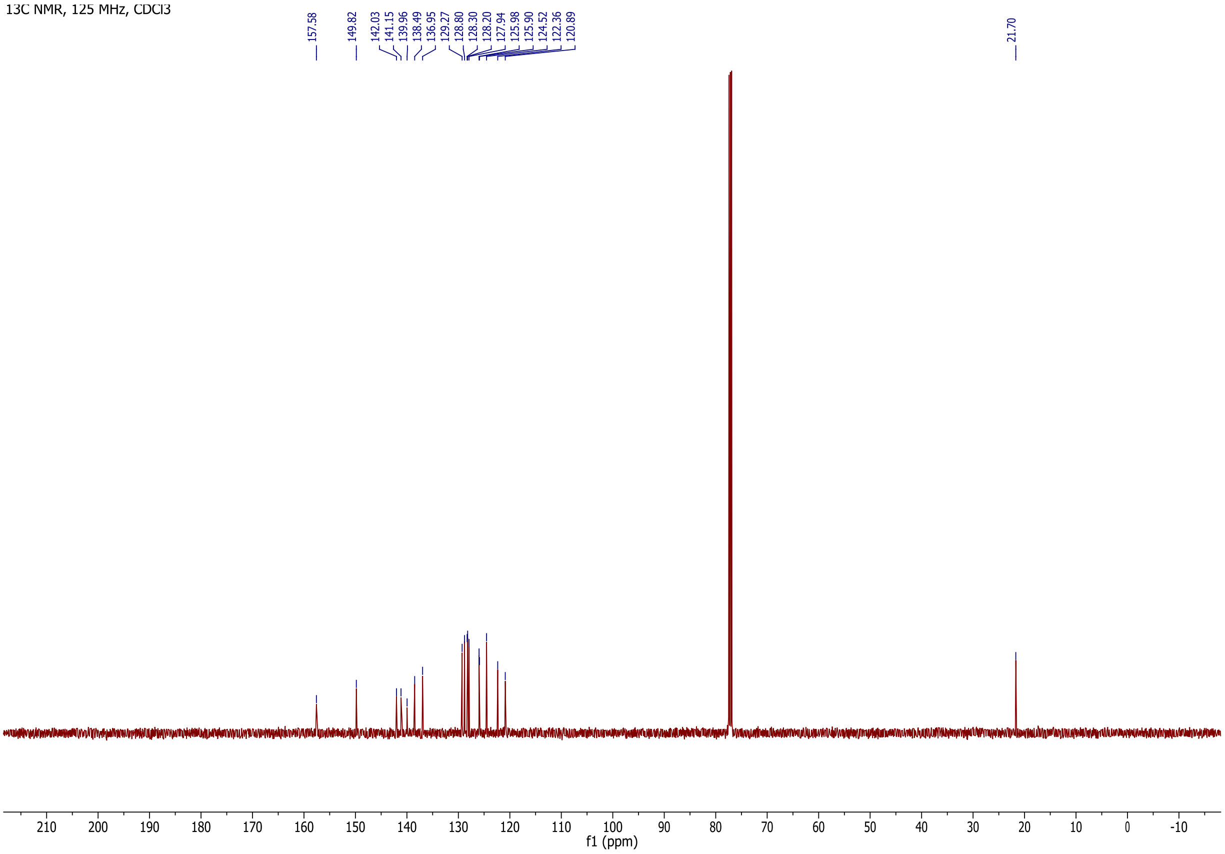

ethyl (E)-3-(3-(pyridin-2-yl)phenyl)acrylate

1H NMR, 400 MHz, CDCl3

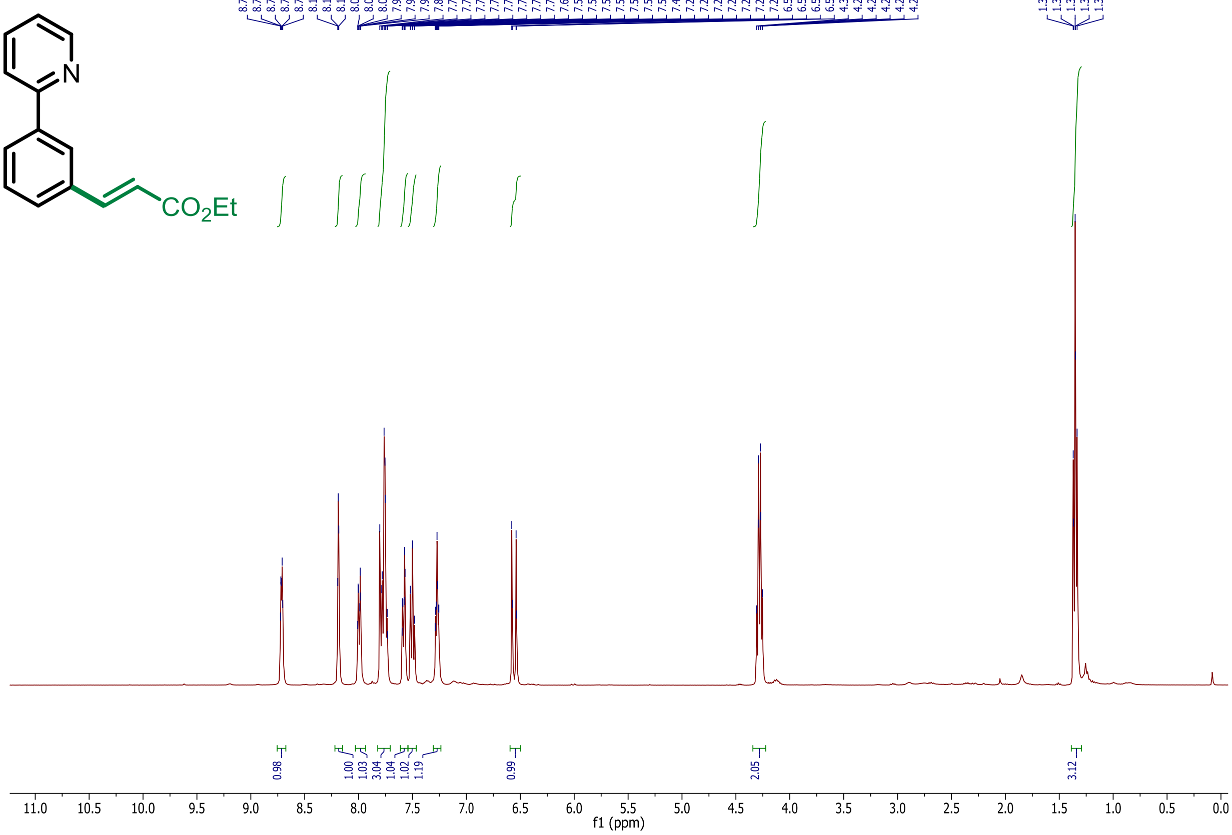

13C NMR, 100 MHz, CDCl3

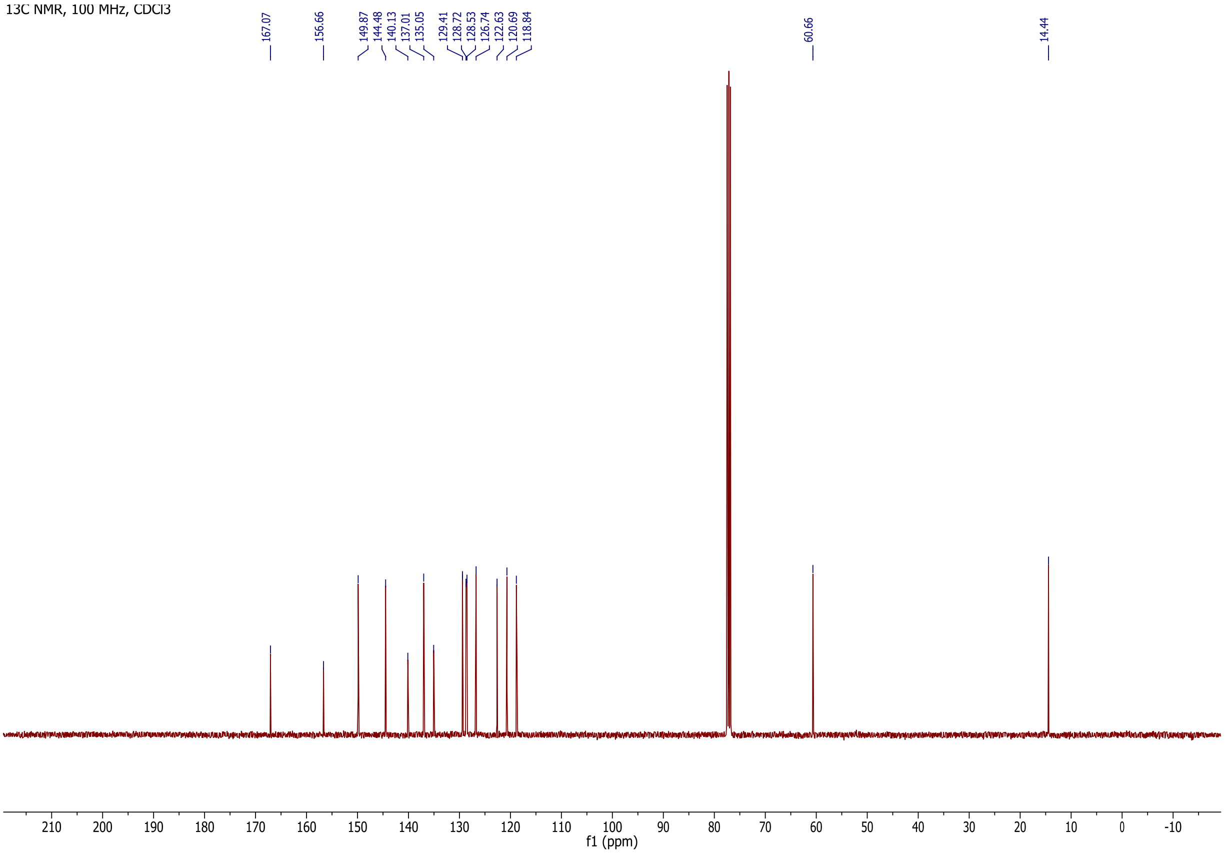

4-(3-(pyridin-2-yl)phenyl)butan-2-one

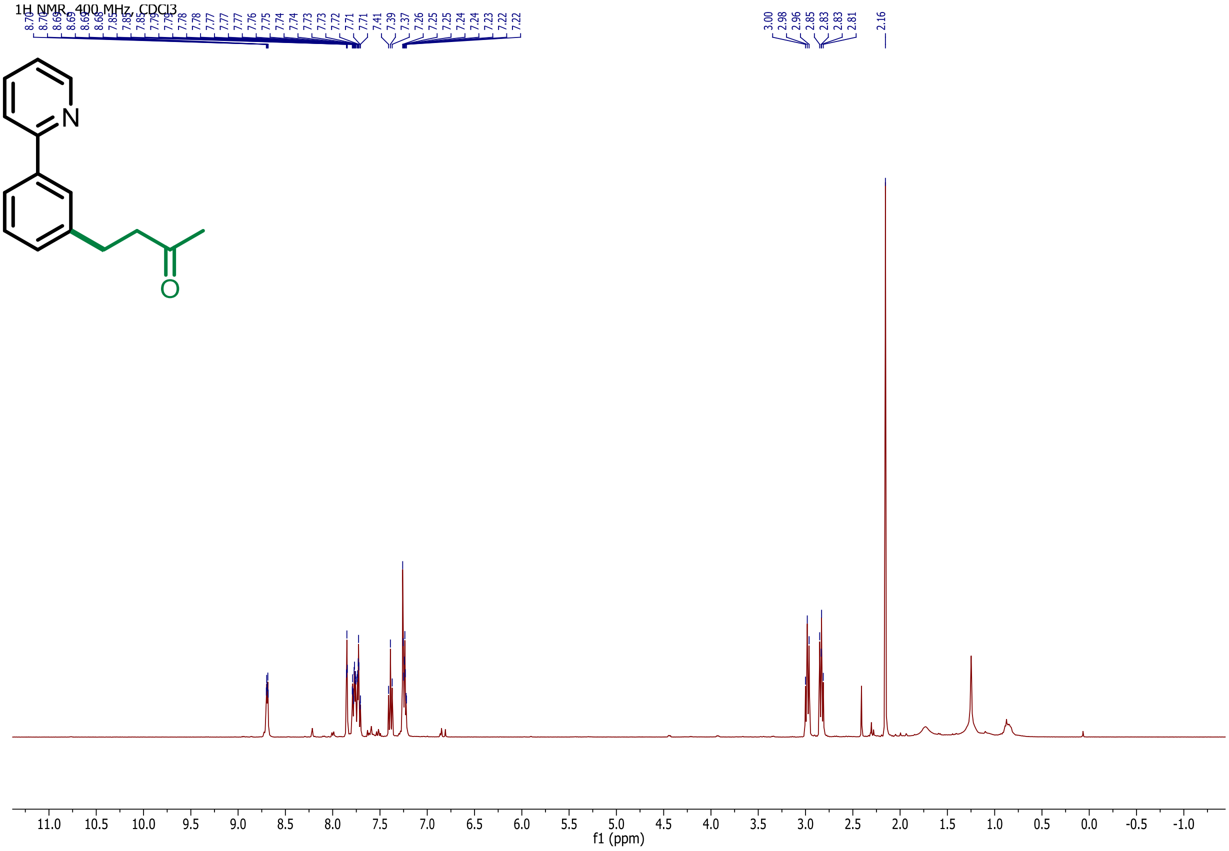

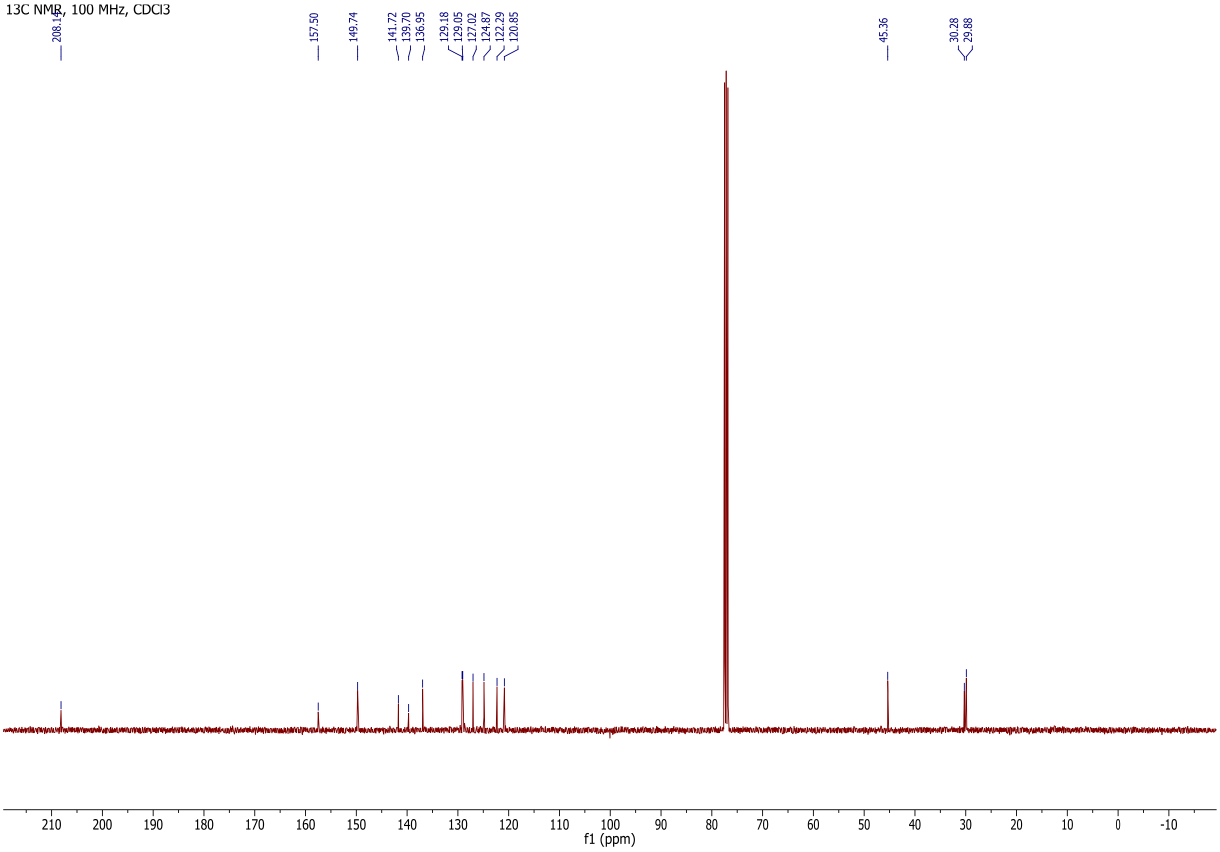

(E)-2-(3-styrylphenyl)pyridine

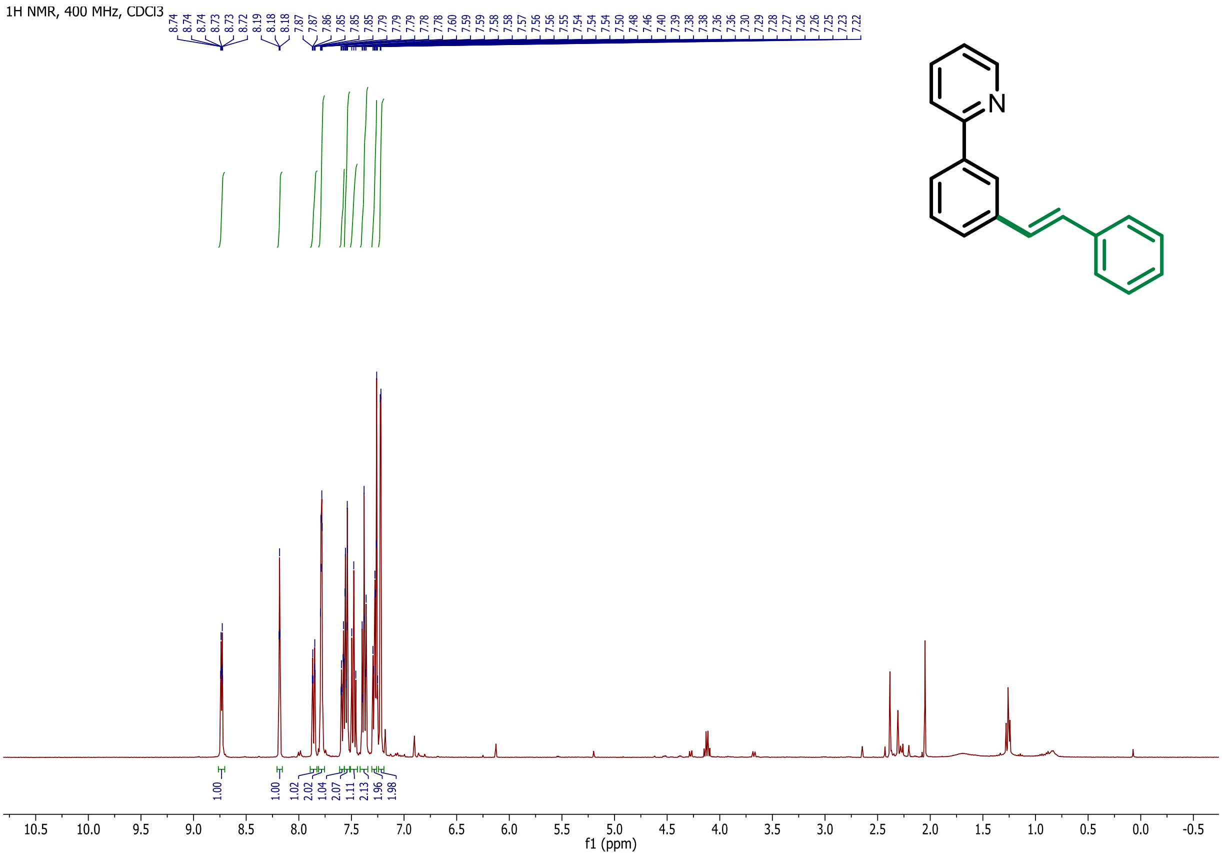

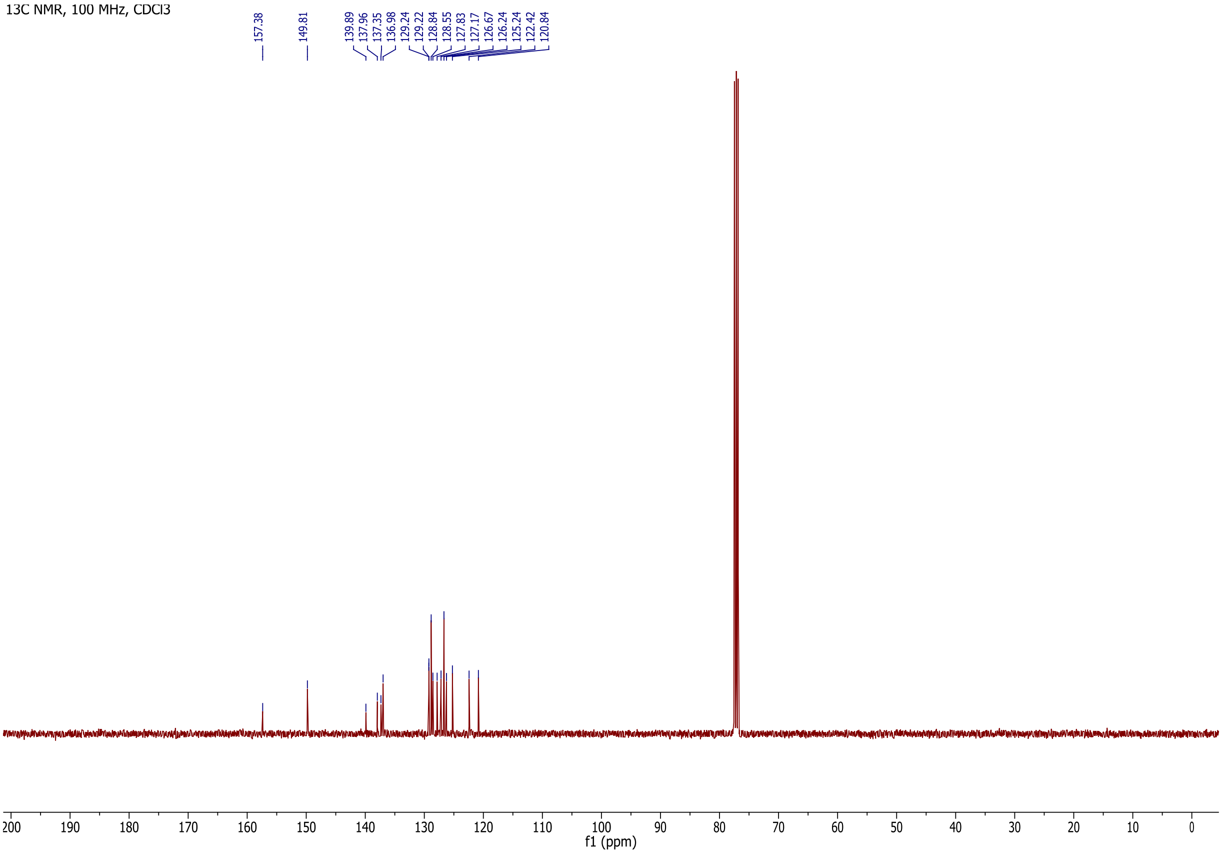

2-(3-bromophenyl)piperidine

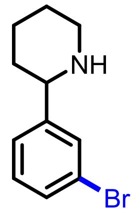

1H NMR, 400 MHz, CDCl3

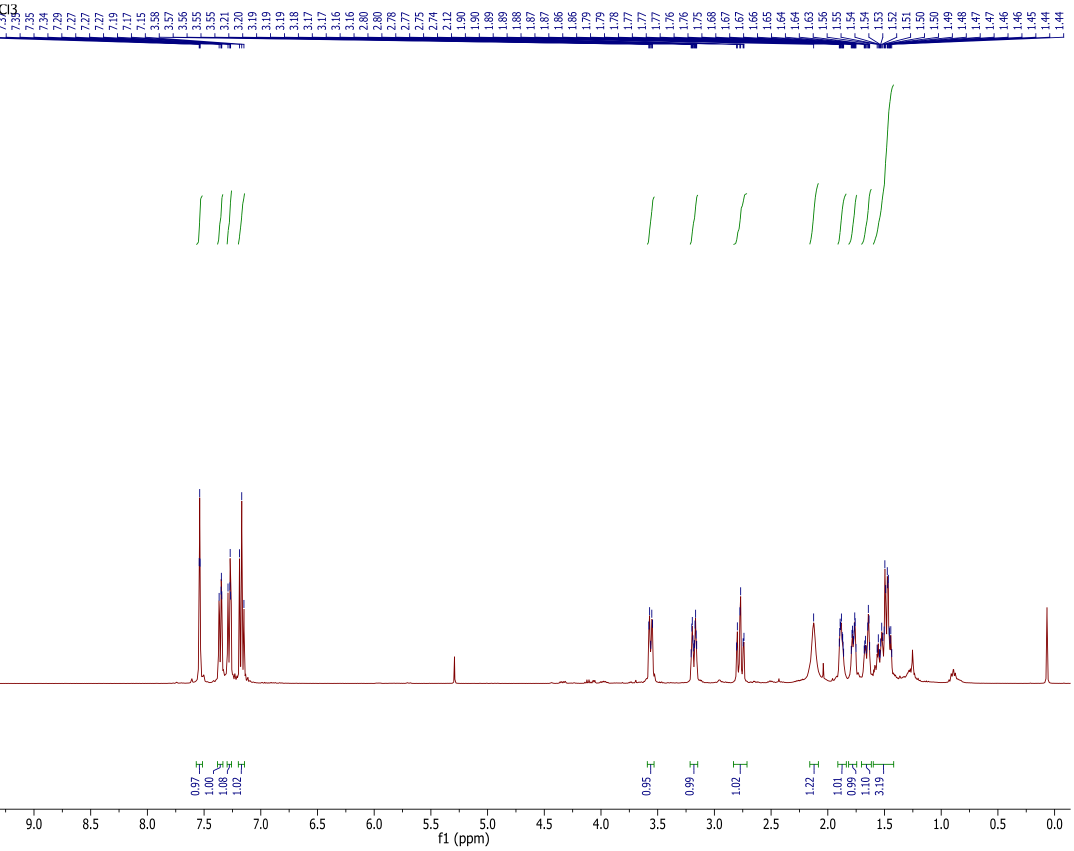

13C NMR, 100 MHz, CDCl3

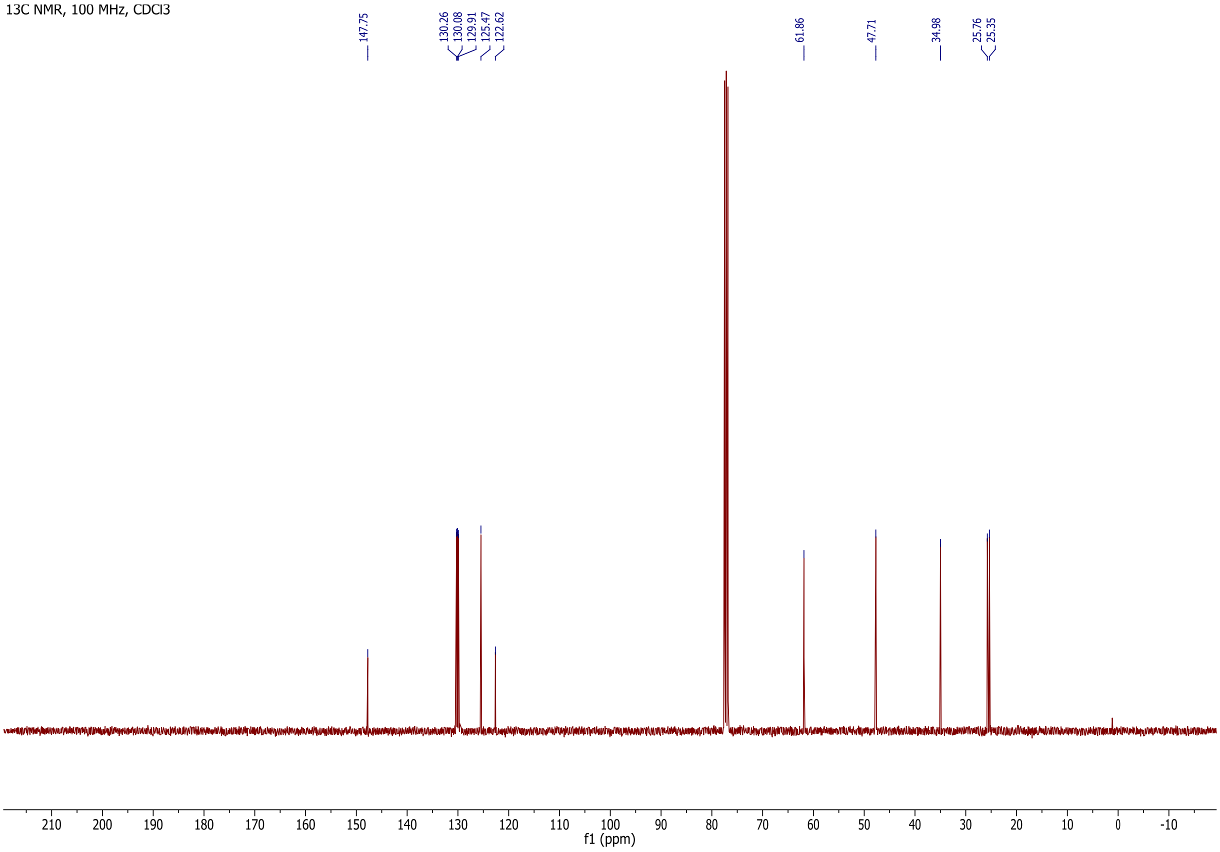

Supplement: Supplementary file 1 [file anie0054-11677-sd1.pdf]
